# HP1 Recruitment in the Absence of Argonaute Proteins in *Drosophila*

**DOI:** 10.1371/journal.pgen.1000880

**Published:** 2010-03-12

**Authors:** Nellie Moshkovich, Elissa P. Lei

**Affiliations:** 1 Laboratory of Cellular and Developmental Biology, National Institute of Diabetes and Digestive and Kidney Diseases, National Institutes of Health, Bethesda, Maryland, United States of America; 2 The Graduate Program in Molecular and Cell Biology, University of Maryland, College Park, Maryland, United States of America; Massachusetts General Hospital, Howard Hughes Medical Institute, United States of America

## Abstract

Highly repetitive and transposable element rich regions of the genome must be stabilized by the presence of heterochromatin. A direct role for RNA interference in the establishment of heterochromatin has been demonstrated in fission yeast. In metazoans, which possess multiple RNA–silencing pathways that are both functionally distinct and spatially restricted, whether RNA silencing contributes directly to heterochromatin formation is not clear. Previous studies in *Drosophila melanogaster* have suggested the involvement of both the *AGO2*-dependent endogenous small interfering RNA (endo-siRNA) as well as Piwi-interacting RNA (piRNA) silencing pathways. In order to determine if these Argonaute genes are required for heterochromatin formation, we utilized transcriptional reporters and chromatin immunoprecipitation of the critical factor Heterochromatin Protein 1 (HP1) to monitor the heterochromatic state of piRNA clusters, which generate both endo-siRNAs and the bulk of piRNAs. Surprisingly, we find that mutation of *AGO2* or *piwi* increases silencing at piRNA clusters corresponding to an increase of HP1 association. Furthermore, loss of piRNA production from a single piRNA cluster results in genome-wide redistribution of HP1 and reduction of silencing at a distant heterochromatic site, suggesting indirect effects on HP1 recruitment. Taken together, these results indicate that heterochromatin forms independently of endo-siRNA and piRNA pathways.

## Author Summary

One role for silent heterochromatin is to preserve the integrity of the genome by stabilizing regions rich in repetitive sequence and mobile elements. Compaction of repetitive sequences by heterochromatin is needed to prevent genome rearrangement and loss of genetic material. Furthermore, uncontrolled movement of mobile elements throughout the genome can result in deleterious mutations. In fission yeast, one important mechanism of heterochromatin establishment occurs through RNA interference, an RNA–dependent gene silencing process. However, it is unclear whether a direct role for RNA silencing in heterochromatin formation is conserved throughout evolution. In the fruit fly, *Drosophila melanogaster,* which harbors multiple RNA–silencing pathways that are both functionally distinct and spatially restricted, previous studies have suggested the involvement of the endogenous small interfering RNA (endo-siRNA) and Piwi-interacting RNA (piRNA) pathways in heterochromatin formation. These small RNA silencing pathways suppress the expression of mobile elements in the soma or in both somatic and germline tissues, respectively. Utilizing complementary genetic and biochemical approaches, we monitored the heterochromatin state at discrete genomic locations from which both types of these small RNAs originate in endo-siRNA or piRNA pathway mutants. Our results indicate that heterochromatin can form independently of these two small RNA silencing pathways.

## Introduction

In *D. melanogaster*, an estimated one-third of the genome is composed of repetitive and noncoding sequences associated with a condensed form of chromatin known as heterochromatin. Heterochromatin is characterized by repeat-rich sequences, hypoacetylation of histone tails, and dimethylation of histone H3 on lysine 9 (H3K9me2) [1]. A conserved nonhistone Heterochromatin Protein 1 (HP1) is a critical component of heterochromatin, localizing predominantly at and near centromeres but also residing at telomeres, and the Y and fourth chromosomes. These regions tend to be rich in transposable elements (TEs), which must be suppressed in order to maintain genomic stability but can serve a cellular function, particularly in the case of *Het-A* and *TART* at the telomeres (reviewed in [2]).

The phenomenon of position-effect variegation (PEV) provided the first glimpse into the role of heterochromatin in gene silencing in *Drosophila*. When a normally euchromatic gene is relocated near heterochromatin, variegated expression results from variable levels of heterochromatin spreading over the gene in each cell. Screens for dominant mutations that either suppress {*Suppressor of variegation* [*Su(var)*]} or enhance {*Enhancer of variegation* [*E(var)*]} PEV were performed to identify key components of heterochromatin. For example, mutation of *Su(var)3-9*, which encodes an H3K9 methyltransferase, was identified in a large screen for modifiers of PEV [3]. Accordingly, loss of HP1, encoded by *Su(var)2-5*, causes increased expression of a gene subject to PEV while an extra copy has the reverse effect [4].

Pioneering genetic and biochemical studies in *Schizosaccharomyces pombe* have shed considerable light on mechanisms of heterochromatin assembly. The RNA interference (RNAi) machinery was found to play a key role in heterochromatin formation by detecting the transcription of specific DNA repeats located at the mating type locus and the centromere and subsequently nucleating heterochromatin. For example, double-stranded RNAs (dsRNA) produced by bidirectional transcription of pericentromeric repeats are processed by the RNase III endonuclease Dicer1 into short interfering RNAs (siRNAs) [5]. The Argonaute1 PAZ and PIWI domain protein binds these siRNAs as part of the RNA-induced transcriptional silencing complex (RITS) [6]. Loading of RITS with siRNA and recruitment of the complex to the site of dsRNA transcription requires the Clr4 histone methyltransferase, which methylates H3K9 [7]. This methylation mark serves as a binding site for Swi6, a fission yeast homolog of HP1, leading to heterochromatin establishment and spreading. Importantly, heterochromatin can also be nucleated independently of RNAi by other mechanisms. For example, in the absence of RNAi the ATF/CREB stress-activated proteins promote heterochromatin formation at the mating type locus [8], and the Taz1 protein can establish HP1 recruitment to telomeres [9]. These studies exemplify the redundancy of RNAi and additional mechanisms with respect to the formation of heterochromatin.

All RNA silencing pathways are characterized by the activity of an Argonaute effector protein that binds directly to small RNA. The five Argonautes in *Drosophila* can be divided into two families based on homology. The AGO subfamily includes AGO1 and AGO2, and the Piwi subfamily consists of Piwi, Aubergine (Aub), and AGO3 (reviewed in [10]). *AGO1* and *AGO2* are expressed throughout the fly while *piwi*, *aub*, and *AGO3* are expressed mainly, although not exclusively, in the gonad [11]–[13]. AGO1 is required for the microRNA pathway, which regulates mRNA expression and functions chiefly through translational repression. Protecting against exogenous double stranded RNA, AGO2 associates with 21–22 nt siRNA produced by Dicer-2 (Dcr-2), and this pathway is required for viral immunity and a robust RNAi response [14],[15]. In addition, AGO2 also binds endogenous siRNAs (endo-siRNAs), the majority of which silence the expression of TEs outside of the gonad [16]–[19].

Suppression of TEs is especially imperative in the gonad in order to limit the propagation of unwanted mutations and is achieved principally by the activity of the Piwi subfamily proteins. Piwi, Aub, and AGO3 bind to 23–30 nt RNAs termed Piwi-interacting RNAs (piRNAs) that are predominantly derived from genomic locations termed piRNA clusters [20],[21]. These piRNA producing loci are mainly pericentromeric and enriched in transposon sequences. From these and previous studies, it became clear that the piRNA pathway exists to eliminate TE transcripts in the gonad [22]–[24]. Based on comparative sequence analysis of piRNAs immunopurified from the ovary, the “ping-pong” or “amplification loop” model for germline piRNA biogenesis was proposed [20]–[23]. Precursor transcripts from piRNA clusters, derived from either one or both strands [25], give rise to piRNAs bound by Piwi, Aub, or AGO3. Those piRNAs antisense to a homologous TE transcript can result in its cleavage, and this event defines the 5′ end of a secondary piRNA that can then bind and cleave an antisense piRNA cluster transcript, and the cycle can continue. Piwi appears to play a minor role in ping-pong piRNA amplification [25],[26], which is thought to occur primarily in the cytoplasmic nuage where Aub and AGO3 localize [20],[23],[27]. In contrast, Piwi resides in the nucleus [28]. Production of precursor transcripts at certain piRNA clusters that give rise to piRNAs from both sense and antisense strands (dual-strand clusters) is dependent on the germline specific HP1 homolog Rhino [29]. Rhino functions specifically in the ping-pong pathway, acting upstream of Aub and AGO3 but not Piwi.

Piwi independently serves an additional role in the silencing of certain TEs expressed in somatic follicle cells surrounding the ovary. This somatic piRNA pathway depends on Piwi alone and therefore does not undergo ping-pong amplification [25],[26],[30]. The *flamenco (flam)* piRNA cluster, which controls the *gypsy*, *ZAM*, and *Idefix* retrotransposons [31],[32], is one of the major sites of primary piRNA production [25],[26],[30],[33]. Piwi associates with piRNAs generated by *flam* and other piRNA clusters and has been proposed to cleave homologous TE transcripts using its Slicer activity [22].

Previous studies suggest that one or more RNA silencing pathways may participate in transcriptional TE silencing by inducing heterochromatin formation. First, mutation of *AGO2* results in pleiotropic cellular defects in early embryos including mislocalization of HP1 and the histone H3 variant CID, which binds specifically the centromere [34]. Later in development, *AGO2* mutants display mislocalization of HP1 on polytene chromosomes of the larval salivary gland [35]. Additionally, silencing of a pericentromeric transcriptional reporter is relieved when the maternally derived pool of AGO2 is reduced. Despite these defects, *AGO2* mutant flies develop normally and are fertile, suggesting that these defects are mild and can be compensated by other mechanisms.

Several pieces of evidence implicate piRNA pathways in establishment or maintenance of heterochromatin in the soma. First, mutation of *piwi*, *aub*, or *spn-E*, encoding an RNA helicase required for the germline piRNA pathway [24],[26], results in defects in heterochromatic silencing and visible changes in heterochromatin localization. These mutants reduce silencing of pericentromeric transcriptional reporters and exhibit mislocalization of HP1 and H3K9me2 in salivary gland polytene chromosomes [36]. Moreover, a recent study identified HP1 as an interactor of Piwi in yeast two-hybrid screens [11]. The two proteins coimmunoprecipitate from embryonic nuclear lysate and display partially overlapping localization patterns in polytene chromosomes. Furthermore, both proteins associate specifically with the chromatin of two transposable elements, *1360* and the *F element*. Based on their findings, the authors propose that Piwi could serve as a recruitment platform for HP1 binding. This model appears not to be applicable to the 3R-TAS subtelomeric region, a site of Piwi chromatin association and piRNA production [21]. Mutation of *piwi* results in an increase of HP1 association and an increase of transcriptional silencing at 3R-TAS. It remains an open question whether other sites in the genome could serve as Piwi-dependent HP1 recruitment sites.

In other metazoans, it is similarly unclear whether RNA silencing can establish heterochromatin directly. A recent study in chicken indicates that a 16 kb constitutive heterochromatin domain that separates the folate receptor gene and the β-globin locus is maintained by a Dicer and Argonaute 2 (cAgo2) dependent mechanism [37]. Intriguingly, cAgo2 was shown to associate with the heterochromatic domain by chromatin immunoprecipitation (ChIP) suggesting a direct effect. However, it is not known whether this represents a general mechanism to maintain heterochromatin.

In this study, we investigated whether HP1 association with heterochromatin in *Drosophila* is mediated by either the *AGO2* dependent endo-siRNA pathway or by *piwi* dependent piRNA pathways. Using transcriptional reporters and ChIP, we show that piRNA clusters are subject to heterochromatic silencing and bound by HP1. Interestingly, mutation of *AGO2*, *piwi* or *aub* results in increased silencing at piRNA clusters and an increase in HP1 association with these loci. Furthermore, loss of piRNA production at a single piRNA locus results in global redistribution of HP1 and a reduction of silencing at a distant heterochromatic site. Therefore, our results indicate that HP1 can associate with chromatin independently of both endo-siRNA and piRNA pathways.

## Results

### Heterochromatin-dependent transcriptional silencing at piRNA clusters

We sought to determine if HP1 is recruited to heterochromatin by AGO2 or Piwi. The majority of genomic regions that produce the bulk of piRNA, termed piRNA clusters, are pericentromeric and rich in transposable elements [20],[21]. These regions also produce endo-siRNA [16]–[19], and due to their proximity to the centromere, may be heterochromatic and serve as platforms for Argonaute mediated HP1 recruitment.

**Figure 1:**
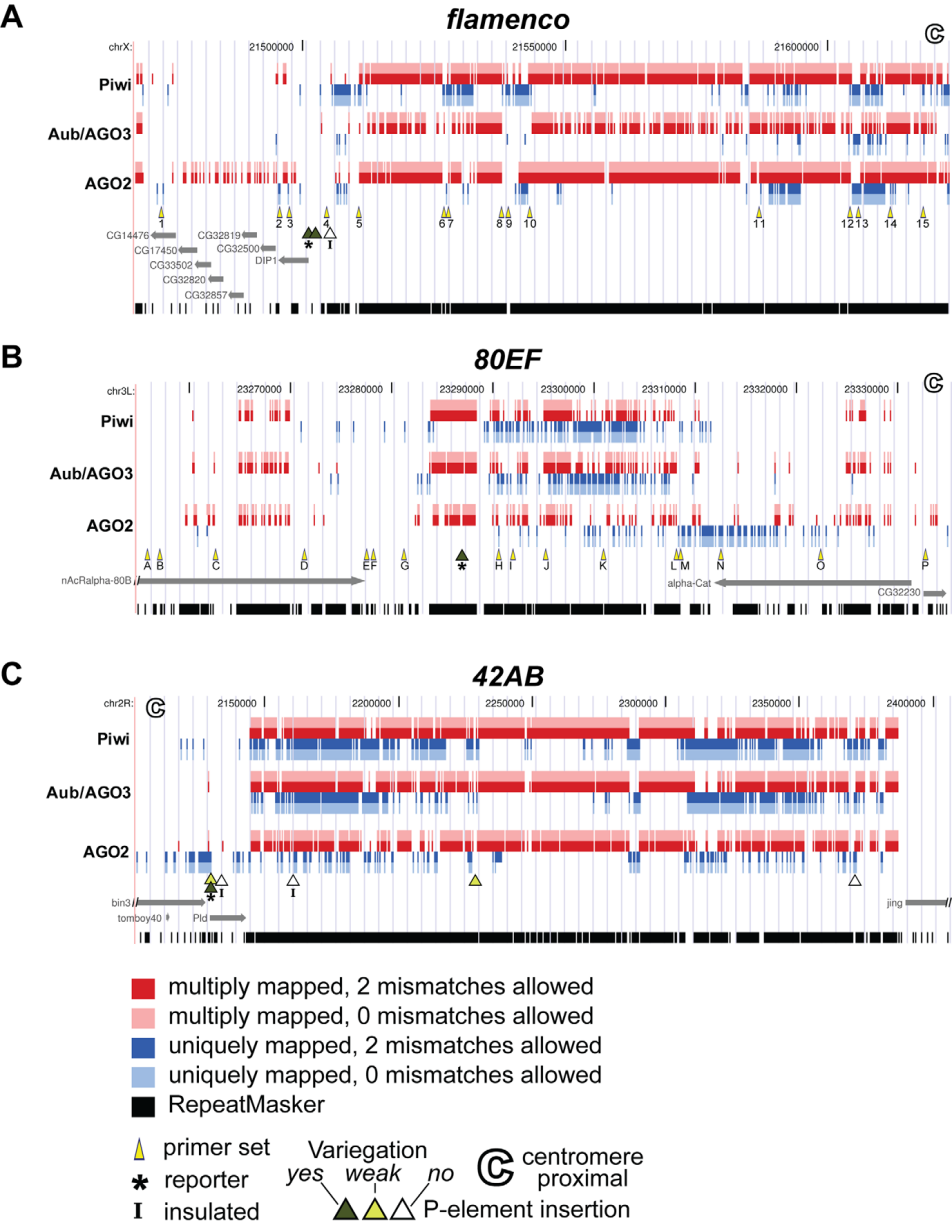
Schematic representation of three top piRNA clusters. Genomic locations of small RNAs, primer sets used for ChIP, and P element insertions at (A) *flam* piRNA cluster on chromosome X, (B) *80EF* piRNA cluster on chromosome 3L, and (C) *42AB* piRNA cluster on chromosome 2R. Sequence datasets derived from previous studies were mapped to the genome using Bowtie software allowing two or zero mismatches [57]. Piwi-immunoprecipitated [20],[21], Aub or AGO3-immunoprecipitated [20] and AGO2-immunoprecipitated [16],[17] reads mapping to multiple locations in the genome are indicated in red (with two mismatches allowed) and pink (with zero mismatches allowed) while uniquely mapping reads are in dark blue (with two mismatches allowed) and light blue (with zero mismatches allowed). Primer sets used for ChIP analysis are indicated by yellow arrowheads. Strongly variegating (dark green triangle), weakly variegating (light green triangle), and non-variegating P elements with high expression levels (white triangle) are indicated. (A) *P{EPgy2}DIP1^EY02625^*, (B) *PBac{PB}c06482*, and (C) *P{EPgy2}EY08366* P element insertions are marked by an asterisk. *SUPor-P* P elements containing insulator sequences are marked by an “I”. Centromere proximal end is marked by a hollow C. RepeatMasker detected sequences are represented in black.

**Figure 2:**
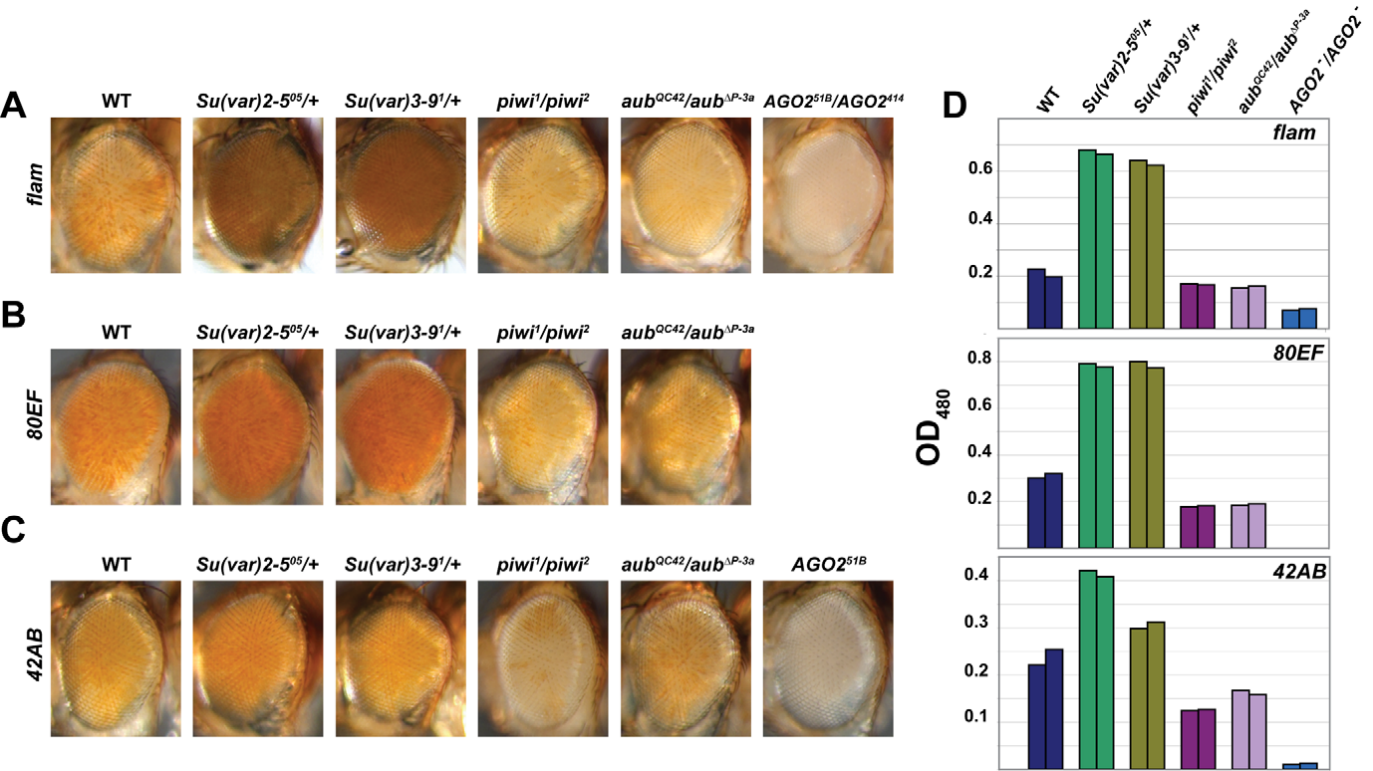
piRNA and endo-siRNA pathway mutants display increased silencing of transcriptional reporters at or near piRNA clusters. Adult eyes of wild type, *Su(var)2-5^05^/+*, *Su(var)3-9^1^/+*, *piwi^1^/piwi^2^*, *aub^QC42^/aub^ΔP-3a^*, and/or *AGO2* ^-^/*AGO2* ^-^ mutants carrying a *mini-white* transgene inserted inside or in close proximity to the (A) *flam*, (B) *80EF*, and (C) *42AB* piRNA clusters. *AGO2^51B^*/*AGO2^414^* mutants are examined in (A) while *AGO2^51B^* mutants are examined in (C). (D) Levels of eye pigment measured at 480 nm extracted from male heads of the indicated genotypes (A–C).

In order to test genetically whether pericentromeric piRNA clusters are heterochromatic, we examined a collection of fly lines bearing P element transgene insertions inside or in close proximity to four piRNA producing loci, *flam*, *80EF*, *42AB*, and *38C*. The P elements contain a *mini-white* transcriptional reporter that was assayed for expression in the adult eye. Genomic locations of these transgene insertions are indicated in relation to previously identified small RNAs immunoprecipitated with Piwi, Aub/AGO3, and AGO2 respectively from various cell types (Figure 1, Figure S1) [16],[17],[20],[21]. Lines harboring P elements inside or in the vicinity of a piRNA cluster exhibit variegating coloration of distinct eye facets similar to PEV, suggesting the presence of variably spreading heterochromatin at their sites of insertion (Figure 2, Table 1). Interestingly, insertions within a piRNA cluster that display high *mini-white* expression without variegation harbor *SUPor-P* constructs, which contain Suppressor of Hairy wing [Su(Hw)] insulator sequences that flank and likely protect the *mini-white* reporter from the effects of surrounding heterochromatin [38].

**Table 1:** Expression of *mini-white* in fly lines harboring P element insertions in four top piRNA clusters.

Insertion	piRNA cluster	Genomic coordinates of insertion	Variegation	Inside piRNA cluster
*P{EPgy2}DIP1^EY02625^*	*flam*	X:21,501,171 [−]	Yes	No
*P{SUPor-P}flam^KG00476^*	*flam*	X:21,505,285 [−]	No	No
*P{GT1}flam^BG02658^*	*flam*	X:21,502,538 [−]	Yes	No
*PBac{WH}CG32230^f00651^*	*80EF*	3L:23,237,018 [+]	Weak	No
*PBac{PB}c06482*	*80EF*	3L:23,286,922 [−]	Yes	Yes
*PBac{PB}CG40470^c06318^*	*80EF*	3L:23,849,420 [+]	No	No
*P{GT1}BG01672^BG01672^*	*42AB*	2R:2,370,529 [−]	No	Yes
*P{EPgy2}EY08366*	*42AB*	2R:2,129,510 [+]	Yes	No
*P{XP}d02126*	*42AB*	2R:2,129,452 [+]	Weak	No
*P{SUPor-P}Pld^KG02714^*	*42AB*	2R:2,133,438 [+]	No	No
*P{SUPor-P}KG09351*	*42AB*	2R:2,160,357 [−]	No	Yes
*PBac{WH}f04291*	*42AB*	2R:2,228,280 [−]	Weak	Yes
*P{EPgy2}EY01034*	*38C*	2L:20,205,306	Yes	Yes
*P{XP}d02757*	*38C*	2L:20,174,988 [+]	Weak	Yes
*PBac{WH}f03348*	*38C*	2L:20,165,746	Weak	Yes
*P{SUPor-P}KG05288*	*38C*	2L:20,166,034 [+]	No	Yes
*PBac{RB}e03575*	*38C*	2L:20,121,359 [+]	Weak	No
*P{SUPor-P}KG02342*	*38C*	2L:20,120,504 [−]	No	No

The genomic coordinates for four top piRNA clusters were defined as previously determined by Brennecke et al., 2007 [20]. The genomic coordinates of the P-element insertions were confirmed by PCR with primers specific to the P-elements and flanking genomic sequences.

Expression analysis of these transcriptional reporter insertions indicates that piRNA clusters and their immediate vicinity are subject to HP1 dependent silencing. Reporter expression levels of three lines harboring an insertion at *flam*, *80EF*, or *42AB* with the most apparent variegation were tested for dependence on heterochromatin. *P{EPgy2}DIP1^EY02625^* is inserted in a gene located on the centromere distal side of the *flam* piRNA producing locus on the X chromosome (Figure 1A), *PBac{PB}c06482* resides within the *80EF* cluster on chromosome 3L (Figure 1B), and *P{EPgy2}EY08366* borders the centromere proximal edge of the *42AB* piRNA locus on chromosome 2R (Figure 1C). In order to test whether these reporters are sensitive to perturbation of heterochromatin, the expression of *mini-white* was examined in *Su(var)2-5^05^*/+ and *Su(var)3-9^1^*/*+* dominant loss-of-function mutants, which are compromised for HP1 and H3K9 methyltransferase activity respectively. As expected, decreased silencing of *mini-white* expression resulting in increased pigmentation was observed for all three insertions in the heterochromatin mutants compared to wild type (Figure 2), suggesting that the vicinity of P element insertion are indeed heterochromatic.

### piRNA and endo-siRNA pathway mutants decrease transcription at piRNA clusters

We next tested whether the transcriptional reporters at piRNA clusters are sensitive to perturbations in the piRNA and endo-siRNA silencing pathways. If Piwi were responsible for direct recruitment of HP1 to piRNA clusters, mutation of *piwi* should increase *mini-white* expression similarly to disruption of heterochromatin. Surprisingly, *piwi^1^*/*piwi^2^* loss-of-function mutants exhibit a substantial loss of reporter expression indicating increased silencing when compared to wild type (Figure 2). Furthermore, *aub^QC42^/aub^ΔP-3a^* loss-of-function piRNA pathway mutants result in a similar reduction of *mini-white* expression. Strikingly, the *flam* transcriptional reporter expression level was decreased dramatically in the transheterozygous endo-siRNA pathway mutant, *AGO2^51B^*/*AGO2^414^* compared to wild type (Figure 2A). Similarly, in the *AGO2^51B^* null mutant, the *42AB* transcriptional reporter displays almost complete silencing (Figure 2C). Spectroscopic analysis of extracted eye pigment verifies the overall changes in *mini-white* expression levels for each genotype compared to wild type (Figure 2D). Additionally, examination of *Dcr-2^L811fsX^* mutants shows a similar mild increase in silencing for the transcriptional reporter inserted near *flam* (Figure S2A). The opposite effects of piRNA and endo-siRNA pathway mutations compared to heterochromatin mutations suggest that these RNA silencing pathways may actually oppose heterochromatin formation at piRNA clusters.

### HP1 chromatin association is increased at piRNA clusters in somatic tissues of RNA silencing mutants

In order to further examine the heterochromatic nature of piRNA clusters at higher resolution, ChIP assays were performed in adult heads to assess HP1 association with two piRNA clusters, *flam* and *80EF*, in the soma. Genomic locations of primer sets that uniquely amplify regions spanning these piRNA clusters are indicated in Figure 1A and 1B. As positive controls, primers for two transposable elements known to recruit HP1, *TART,* a telomere-specific non-LTR retrotransposon, and *1360,* a DNA transposon were also tested [39]–[40]. Euchromatic genes *hsp26* and *yellow* were also included in the analysis as negative controls for HP1 association.

**Figure 3:**
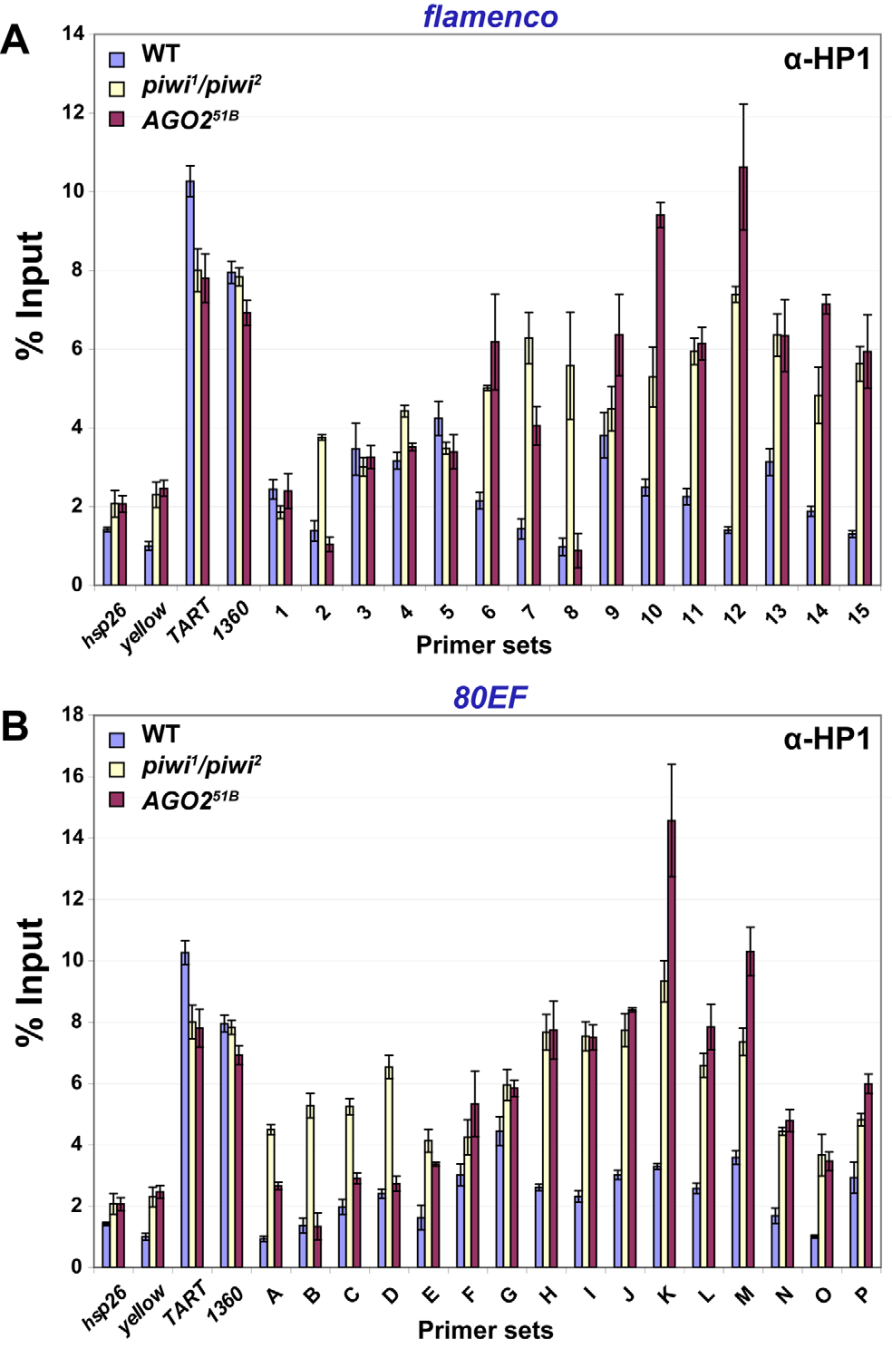
HP1 associates with chromatin at piRNA clusters, and its levels increase in RNA–silencing mutants. ChIP at (A) *flam* and (B) *80EF* piRNA clusters in wild type (blue), *piwi^1^/piwi^2^* (yellow), and *AGO2^51B^* (red) mutants from adult heads with antibodies specific to HP1. Percent input immunoprecipitated is shown for each primer set, and error bars indicate standard deviation of quadruplicate PCR measurements.

In wild type fly heads, HP1 is observed at or near locations that give rise to piRNAs and endo-siRNAs at both *flam* and *80EF* loci. ChIP was performed using α-HP1 antibodies in chromatin prepared from wild type heads, and the amount of DNA associated was determined by quantitative PCR using specific primer sets. As expected, low levels of *hsp26* and *yellow* are immunoprecipitated with HP1, while *TART* and *1360* levels are enriched above the euchromatic genes by over six-fold (Figure 3). At *flam*, HP1 associates with the majority of regions that produce high levels of piRNAs or endo-siRNAs approximately two to three-fold over the euchromatic sites (Figure 3A, primer sets 1–15). Similarly, at *80EF*, HP1 immunoprecipitates piRNA and endo-siRNA producing regions two to three-fold higher than the negative controls indicating the presence of heterochromatic marks at these loci (Figure 3B, primer sets G-M). Regions flanking these areas display approximately one to two-fold enrichment over euchromatic sites, which may be due to tapering of HP1 spreading (Figure 3B, primer sets A-F and N-P). ChIP using antibodies directed against the chromatin insulator protein Su(Hw) verified its presence at known insulator sequences *gypsy* and *1A-2* [41] but only background levels at *TART*, *1360*, and piRNA clusters, indicating the specificity of HP1 association at these sites (Figure S3). Rabbit IgG negative control immunoprecipitations yielded negligible amounts of DNA for all sites tested (<0.3% input).

Consistent with the transcriptional reporter assay, RNA silencing mutants display elevated levels of HP1 at piRNA clusters. ChIP of HP1 was performed in *piwi^1^*/*piwi^2^* mutant heads, and similar levels at positive and negative controls were obtained compared to wild type (Figure 3). In contrast, at the *flam* locus, a two to five-fold increase in HP1 levels is observed at the centromere proximal side of the locus compared to wild type (Figure 3A, primer sets 6–15). Little change in HP1 recruitment is observed at the centromere distal end of *flam* in *piwi^1^*/*piwi^2^* mutants (Figure 3A, primer sets 1–5). At *80EF*, HP1 levels increase two to three-fold in *piwi^1^*/*piwi^2^* mutants compared to wild type across all primer sets examined (Figure 3B, primer sets A-P).

In order to address differences in strain background and potential accumulation of TEs in *piwi* mutant strains, we performed ChIP assays comparing *piwi^1^*/*piwi^2^* mutants to a *piwi^1^*/*+* heterozygous strain and obtained similar results (Figure S4).

ChIP experiments performed in *AGO2^51B^* mutant heads show a similar overall increase of HP1 at piRNA clusters compared to *piwi^1^*/*piwi^2^* mutants. Levels of HP1 at *hsp26*, *yellow*, *TART*, and *1360* are similar in *AGO2^51B^* mutants and wild type while differences are apparent at piRNA clusters (Figure 3). At *flam*, *AGO2^51B^* mutants display a two to seven-fold increase of HP1 association with the centromere proximal side compared to wild type (Figure 3A, primer sets 6–15). At the centromere distal end, no significant changes in HP1 levels are detected (Figure 3A, primer sets 1–5). For *80EF*, *AGO2^51B^* mutants show similar levels of HP1 to wild type at the centromere distal end (Figure 3B, primer sets A-D) while an approximately two to five-fold increase of HP1 is detected in the remainder of the regions tested (Figure 3B, primer sets E-P). Moreover, ChIP assays in *AGO2^51B^* homozygous mutants compared to an *AGO2^51B^*/+ heterozygous strain produced similar results (Figure S5). Similar to *AGO2^51B^* mutants, *Dcr-2^L811fsX^* mutants show an increase of HP1 at regions that produce small RNAs compared to wild type (Figure S2B and S2C). HP1 protein levels in wild type, *piwi^1^/piwi^2^*, and *AGO2^51B^* fly heads are similar indicating that the increased chromatin association observed is not due to an increased amount of HP1 (Figure S6). The increased HP1 chromatin association with piRNA clusters in RNA silencing mutants compared to wild type is consistent with increased silencing of P element insertions, and these results suggest that at least some of the observed effects on reporter gene expression in RNA silencing mutants are due to chromatin related events. Taken together, these data suggest an antagonistic effect of Piwi, Aub, and AGO2 on HP1 recruitment to chromatin in somatic tissue.

### HP1 also associates with piRNA clusters in ovaries

**Figure 4:**
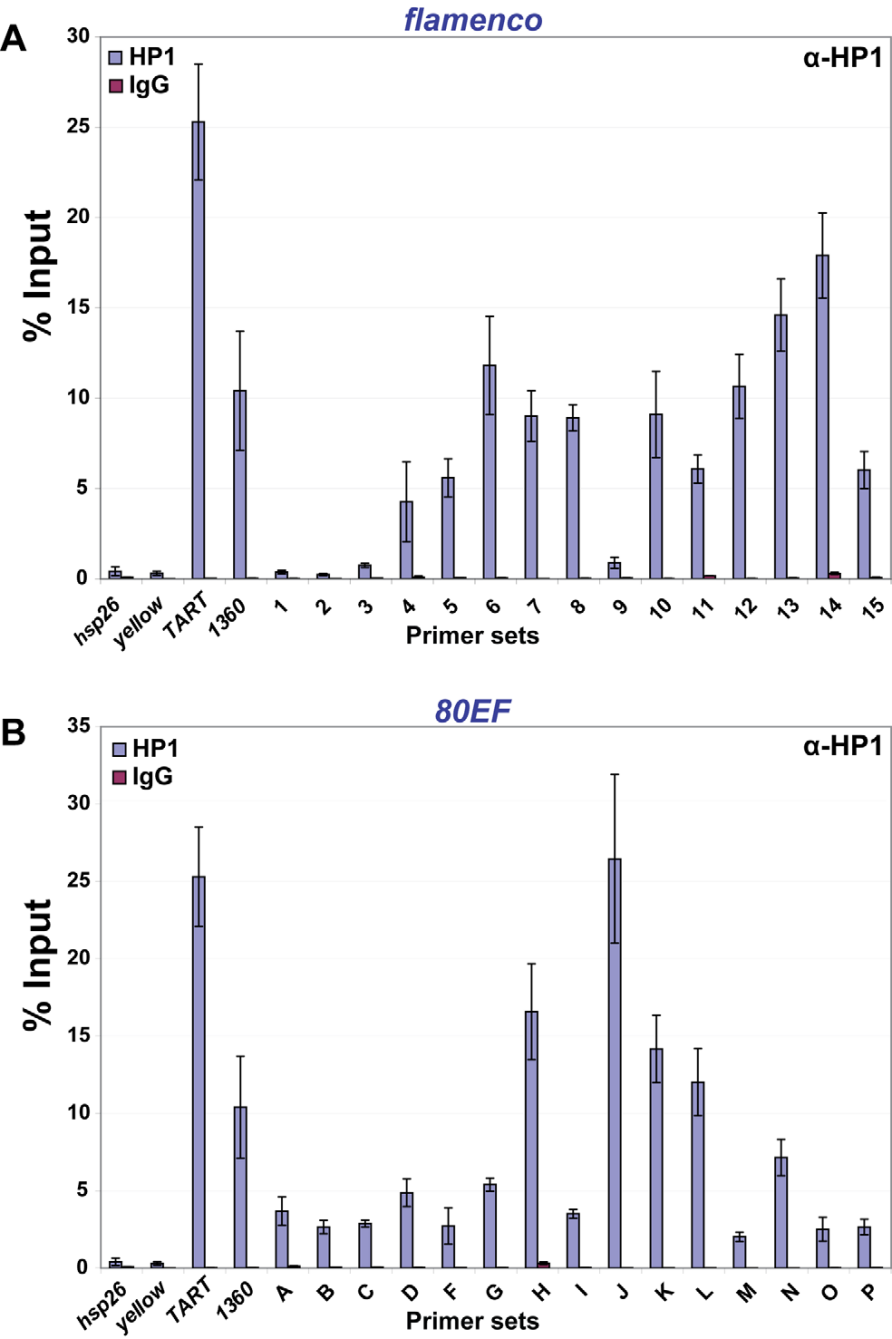
HP1 associates with chromatin at piRNA clusters in ovaries. ChIP at (A) *flam* and (B) *80EF* piRNA clusters in wild type ovaries with antibodies specific to HP1 (blue) and normal rabbit IgG (red).

Given the evidence that transposable elements are mainly silenced in the gonad via piRNA pathways and in the soma via the endo-siRNA pathway, we wanted to determine whether HP1 also associates with piRNA clusters in gonadal tissues. Therefore, we investigated HP1 recruitment to piRNA clusters in wild type ovaries by ChIP. As in heads, low levels of *hsp26* and *yellow* are immunoprecipitated with HP1, whereas *TART* and *1360* levels are enriched above the euchromatic genes by over ten-fold (Figure 4). At the *flam* locus, a four to fifteen-fold increase over the euchromatic sites in HP1 levels is observed at most sites at the centromere proximal side of the locus (Figure 4A, primer sets 4–15). Similarly, at *80EF*, HP1 immunoprecipitates small RNA producing regions two to twenty-fold higher than euchromatic sites indicating the presence of heterochromatic marks at these loci (Figure 4B, primer sets A-P). Rabbit IgG negative control immunoprecipitations yielded negligible amounts of DNA for all sites tested. We were unable to immunoprecipitate DNA at levels above background from either heads or whole ovaries using multiple antibodies to Piwi, Aub, AGO3, and AGO2 that have been used in previous studies for immunoprecipitation or immunofluorescence (data not shown) [11],[22],[23],[42].

### HP1 chromatin association is not affected greatly by depletion of Piwi in somatic ovarian follicle cells

**Figure 5:**
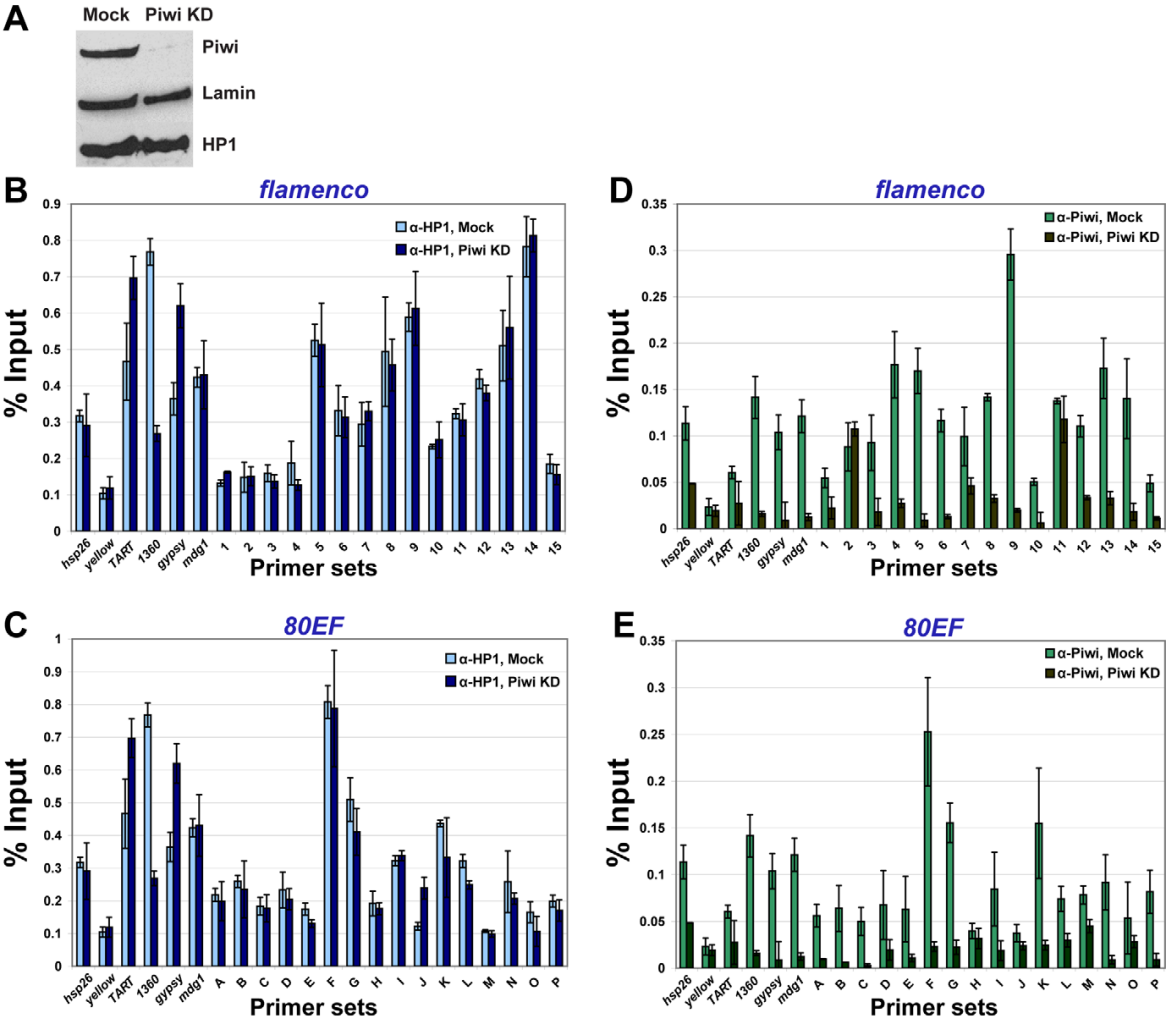
Depletion of Piwi from ovarian somatic follicle cells does not affect HP1 recruitment to piRNA clusters. (A) Western blotting of Piwi, HP1 and Lamin in OSC cells that were either mock treated (left lane) or treated with siRNA directed against *piwi* (right lane, Piwi KD). ChIP at *flam* (B,D) and *80EF* (C,E) piRNA clusters in mock treated and Piwi KD OSC cells with antibodies specific to HP1 (B,C) or Piwi (D,E).

We wished to address whether HP1 association with piRNA clusters is dependent on Piwi in the gonad, which express high levels of both proteins. Due to a complete loss of germ cells and the severe underdevelopment of ovary tissue in *piwi* mutants, it was not possible to obtain enough mutant material to perform ChIP. Therefore, we examined the recruitment of HP1 to chromatin in an ovarian somatic follicle cell line (OSC) that expresses Piwi but not Aub or AGO3 and produces only primary piRNAs, a large proportion of which derive from the *flam* locus [30]. The majority of Piwi was depleted from OSC cells by siRNA-mediated knockdown, and depletion of Piwi does not affect HP1 or Lamin protein levels compared to mock transfected cells (Figure 5A).

Subsequently, we investigated HP1 recruitment to piRNA clusters by ChIP in OSC cells. In mock treated cells, low levels of *hsp26* and *yellow* are immunoprecipitated with HP1, while *TART* and *1360* levels are enriched above the euchromatic genes by 1.5- to over two-fold (Figure 5B and 5C). Two additional TEs tested, *gypsy* and *mdg1*, are immunoprecipitated at similar levels to *TART* with HP1 (Figure 5B–5E). At *flam*, HP1 associates with the piRNA cluster similar to TE levels (Figure 5B and 5C). Despite much lower piRNA production from the *80EF* cluster in OSC compared to *flam* [30], HP1 associates with piRNA producing regions of *80EF* at similar levels to *flam* and TEs (Figure 5C, primer sets A-P). Overall, the HP1 recruitment profile in OSC is similar to that of heads and whole ovaries albeit at lower relative levels. In Piwi knockdown cells, no significant differences are seen for HP1 recruitment to all sites compared to mock treated cells except a two-fold decrease at the *1360* element. Rabbit IgG negative control immunoprecipitations yielded low amounts of DNA for all sites tested (<0.06% and <0.07% input for mock and Piwi knockdown cells, respectively).

Importantly, Piwi association with chromatin is detectable in OSC cells, but its profile differs from that of HP1. In mock treated cells, antibodies directed against Piwi [22] immunoprecipitate euchromatic sites at levels similar to that of TEs (Figure 5D and 5E). Furthermore, the majority of regions producing piRNA at *flam* is also immunoprecipitated at comparable levels to both euchromatic sites and TEs (Figure 5D). Moreover, levels of Piwi association with *80EF* is akin to that of *flam*, while several sites in both *flam* and *80EF* clusters show particular enrichment of Piwi up to three-fold compared to the average association with other sites tested (Figure 5D and 5E). In Piwi knockdown cells, Piwi chromatin association drops two to five-fold, down to background levels at all sites except for some residual association with two sites in or near the *flam* locus. Mouse IgG negative control immunoprecipitations yielded low amounts of DNA in comparison to α-Piwi immunoprecipitations in mock treated cells for all sites tested (<0.04% and <0.02% input for mock and Piwi knockdown cells, respectively). We conclude that in ovarian somatic follicle cells, reduction of the total pool of Piwi as well as the chromatin bound fraction does not affect HP1 association with piRNA clusters and has a minimal effect on HP1 association with TE chromatin association.

### Loss of piRNA production from a single cluster results in global HP1 mislocalization

**Figure 6:**
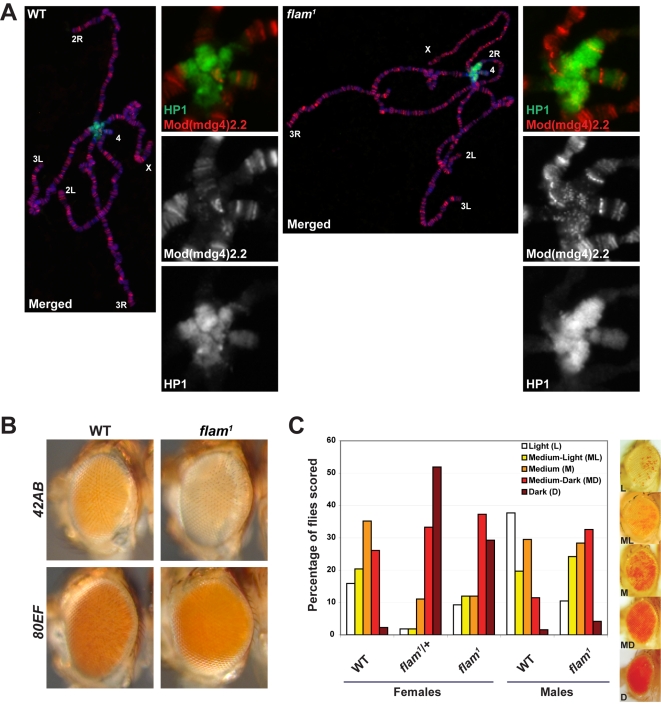
Mutation of the *flam* piRNA cluster results in global HP1 redistribution. (A) Wild type (left) and *flam^1^* (right) polytene chromosomes stained with antibodies directed against HP1 (green) and a reference protein Mod(mdg4)2.2 (red). DNA is stained with DAPI (blue). Chromosome arms are labeled, and insets of the enlarged chromocenter are shown. (B) Adult eyes of wild type and *flam^1^* mutants carrying a *mini-white* transgene inserted in *42AB* (top row) and *80EF* (bottom) piRNA clusters. (C) Degree of eye pigmentation due to expression of the *DX1* transgene array at 50C on chromosome 2L, which undergoes repeat-induced heterochromatic silencing, in wild type, *flam^1^*/+, and *flam^1^* female flies and wild type and *flam^1^* male flies. Scoring of variegation in the eye is categorized into five groups that range between light (few pigmented facets) to dark (almost all pigmented facets). Percentage of flies falling into each category was graphed. Representative eyes are shown on right.

We next sought to determine whether loss of piRNA production at a single piRNA cluster would affect HP1 recruitment to chromatin. Previous studies have shown that mutation of various RNA silencing components results in global mislocalization of HP1 on polytene chromosomes [35]–[36]. Mutation of *flam* has been previously shown to result in loss of piRNA production [20] and upregulation of the *gypsy* retroelement [32]. In order to obtain a genome-wide view of HP1 chromatin association in *flam* mutants, we examined the localization of HP1 to highly replicated salivary gland polytene chromosomes from either wild type or *flam^1^* mutant third instar larvae by indirect immunofluorescence using α-HP1 antibodies. In wild type, HP1 localizes predominantly to a concentration of heterochromatin where the centromeres of each chromosome coalesce, termed the chromocenter (Figure 6A, green). In contrast, *flam^1^* mutants display expansion of HP1 at the chromocenter. Spreading of HP1 is apparent on the second and third chromosomes, but not on the X chromosome, where *flam* is located. As a reference, we also examined the localization of the chromatin insulator protein Mod(mdg4)2.2, which is unchanged in localization between wild type and *flam^1^* (Figure 6A, red). The extent of HP1 chromocenter expansion is comparable to the level of HP1 expansion that we observe in *spn-E^hlsE1^*/*spn-E^hlsE616^* mutants (Figure S7). A lesser degree of HP1 expansion was also observed in *flam^BG02658^/flam^KG00476^* mutants (data not shown). Finally, total HP1 levels are unchanged in *flam^1^* whole flies compared to wild type (Figure S6). These results indicate a global change in HP1 localization resulting from inactivation of a single piRNA cluster.

We reasoned that accumulation of HP1 at the chromocenter of *flam^1^* mutants may result in an increase in silencing at pericentromeric sites. Therefore, the expression of transcriptional reporters at *42AB* or *80EF* piRNA clusters, which are located on different chromosomes from the *flam* locus, was examined in *flam^1^* mutants. Compared to wild type, *flam^1^* mutants harboring a P element insertion at either *42AB* or *80EF* piRNA clusters display mildly decreased pigmentation suggesting increased silencing at these distinct pericentromeric loci (Figure 6B).

### Mutation of the *flam* piRNA cluster suppresses heterochromatic silencing at a distant site

Finally, to verify HP1 genome-wide redistribution in *flam^1^*mutants, we examined the effect of *flam^1^* on the silencing of a centromere distal heterochromatic site on a different chromosome. The *DX1* transgene array consists of seven *mini-white* P elements with one inverted copy at a normally euchromatic site at 50C on chromosome 2R [43]. Due to this configuration, the array forms ectopic repeat induced heterochromatin and displays a variegated phenotype similar to PEV that is dependent on HP1. Expression of the *DX1* array was assessed based on variegation of eye pigmentation in wild type, heterozygous *flam^1^*/+, and homozygous or hemizygous *flam^1^* mutants (Figure 6C). Due to a wide range of eye coloration, variegation was scored by categorization into five groups that ranged between Light (few pigmented facets) to Dark (almost all facets pigmented). For females, 3% of wild type was classified as Dark, while 29% of *flam^1^*/+ and 52% of *flam^1^* mutants displayed the same high level of pigmentation. In males, 15% of wild type was scored as Medium-Dark or Dark while 40% of *flam^1^* males fell into these categories. These results indicate that mutation of *flam* can suppress heterochromatic silencing in *trans*. Taken together with the HP1 centromeric expansion in polytene chromosomes and increased pericentromeric silencing in *flam^1^* mutants, there appears to be a global redistribution of HP1 resulting from the loss of piRNA production from a single locus.

## Discussion

In this study, we tested directly whether the Argonautes AGO2 or Piwi recruit HP1 to chromatin. As candidate sites for Argonaute/HP1 interaction, we examined whether piRNA clusters may be heterochromatic using both genetic and molecular approaches. First, P elements inserted at or near pericentromeric piRNA clusters were assayed as transcriptional reporters, and these transgenes were found to display variegated expression that is increased in heterochromatin mutants. Next, ChIP with α-HP1 antibodies showed that HP1 associates with piRNA clusters at levels significantly above euchromatic sites. However, mutation of *piwi*, *aub*, or *AGO2* leads to a modest increase in silencing of transcriptional reporters as well as an increase of HP1 association at piRNA clusters in heads. In ovarian somatic follicle cells, in which both Piwi and HP1 are highly expressed, depletion of Piwi results in little or no change in HP1 recruitment to piRNA clusters and TEs. Furthermore, loss of piRNA production at a single locus results in expansion of HP1 at the centromere. In these *flam^1^* mutants, silencing of a distant heterochromatic transgene array is reduced, further indicating a global redistribution of HP1 and suggesting indirect effects. Taken together, the results argue against direct recruitment of HP1 or maintenance of its association by AGO2 or Piwi in the soma.

### AGO2 and Piwi are not required for HP1 association at piRNA clusters

Several reasons dictated the choice of piRNA clusters as the focus of our analyses. First, both endo-siRNAs and piRNAs are generated from these loci [16]–[21]. Next, we reasoned that at least some piRNA clusters are likely to be heterochromatic because of their strong bias toward TE-rich pericentromeric positions in the genome [20],[21], in close proximity to the vast majority of HP1 localization. In fact, early cloning attempts determined that the *flam* locus is located in a repetitive, TE rich heterochromatic region [44]. Furthermore, the pericentromeric position of these clusters likely coincides with the transition between euchromatin and heterochromatin, corresponding to the borders of HP1 spreading. This characteristic allows variegation assays, which monitor the variable spreading of HP1 and heterochromatin, to be extremely sensitive. ChIP assays at the borders of HP1 spreading would also likely be optimally sensitive to both local and overall changes in HP1 chromatin association. Finally, piRNA clusters contain enough unique sequence for specific primer design and monitoring by directed ChIP analysis.

Given that *AGO2* is the predominant Argonaute expressed outside the gonad that participates in the silencing of TEs in the soma, we tested whether AGO2 could recruit HP1 to chromatin in somatic tissue. Moreover, it has been shown that *AGO2* mutants exhibit mislocalization of HP1 [34],[35]. However, our results show that mutation of *AGO2* results in a strong increase of silencing of transcriptional reporters at or near piRNA clusters and a mild increase of HP1 chromatin association in heads. Given the extent of increased silencing in the *AGO2* mutant compared to *piwi* or *aub* mutants, which accumulate HP1 on chromatin to a similar degree, a posttranscriptional step of silencing likely contributes to the negative effects observed on transcriptional reporters. *AGO2* mutants show a plethora of cellular defects during early nuclear divisions but develop normally and are fertile suggesting that effects on these various processes as well as HP1 localization are mild or otherwise compensated [34]. Therefore, *AGO2* is unlikely to be required for HP1 recruitment in this tissue.

Additionally, we find that HP1 association at piRNA clusters does not depend on the presence of Piwi. Our analysis of piRNA clusters included *flam*, a primary piRNA cluster, and *80EF*, a germline piRNA producing locus. We examined both *flam* and *80EF* clusters in somatic head tissue and ovaries, which are a mixed population of somatic follicle and germline derived cells. In heads, there is no apparent requirement for *piwi* with respect to HP1 recruitment to the piRNA clusters or to TEs that were examined.

In our study, Piwi chromatin association was detected only in OSC cells, and its presence is dispensable for HP1 chromatin association. The *flam* piRNA cluster produces high levels of primary piRNA in OSC while *80EF* is active for piRNA production in germ cells but not in OSC [25],[26],[30]. Nonetheless, Piwi associates with both the *flam* and *80EF* clusters at comparable levels, suggesting that the amount of piRNA production from a particular locus does not correlate with Piwi chromatin association. Furthermore, the pattern of Piwi chromatin association in OSC differs from that of HP1 in that there is no particular enrichment of Piwi at TEs above euchromatic sites and only a minor accumulation at a few sites in the *flam* and *80EF* piRNA clusters. When Piwi levels were reduced by siRNA knockdown, Piwi chromatin association was essentially abolished but HP1 recruitment was not affected except for a two-fold decrease over the *1360* element. Previous studies suggested that the *1360* element may be responsible for nucleating heterochromatin on the largely heterochromatic fourth chromosome and further showed that mutation of factors representing all RNA silencing pathways, *piwi*, *aub*, *spn-E*, *Dcr-1*, and *Dcr-2*, affect *1360* dependent heterochromatic silencing [40],[45]. Unlike the results in adult heads, no accumulation of HP1 over piRNA clusters was detected as a result of Piwi knockdown in OSC cells. This discrepancy may reflect differential effects in distinct cell types or the length of the Piwi knockdown in OSC cells, which was at least adequate to essentially eliminate Piwi chromatin association. In a related but independently derived ovarian somatic follicle cell line (OSS), Piwi and HP1 do not colocalize in the nucleus [33], and this finding supports the conclusion that Piwi does not direct HP1 recruitment in this cell type. Also consistent with our results, HP1 remains localized to the chromocenter in salivary gland polytene chromosomes in *piwi* null mutants [11],[36]. We conclude that association of HP1 with chromatin can occur independently of *AGO2* and *piwi* in somatic tissue.

A previous study addressed the role of the germline piRNA pathway in HP1 association with transposable elements. The *spn-E* gene controls predominantly germline piRNA production but does not affect the somatic piRNA pathway [26]. ChIP was used to show that *spn-E* mutants display significantly decreased levels of H3K9me3 and HP1 at telomeric *Het-A* but similar to wild type HP1 levels at the *I-element* and *copia* TEs, which are distributed throughout the genome [46]. This modest reduction of HP1 at *Het-A* was apparent in ovaries but not in carcasses, which contain only somatic tissue. One caveat to this study is that ChIP was performed using primers that detect all TEs matching a particular sequence, thus measuring average HP1 and H3K9me levels on TEs across the genome. Nonetheless, this work suggests a limited role for the germline piRNA pathway in HP1 recruitment at the telomere.

### Additional candidate platforms for Piwi-dependent HP1 recruitment

Several studies have shown that Piwi associates with at least some heterochromatic sites in the genome, but direct evidence that any of these sites serve as recruitment platforms for HP1 and subsequent spreading is lacking. The best characterized Piwi-associated site is the heterochromatic 3R-TAS subtelomeric region, which generates the abundant Piwi bound 20nt 3R-TAS piRNA. Surprisingly, the role of *piwi* at this location is transcriptional activation, as *piwi* mutants display increased transcriptional silencing of a nearby reporter transgene as well as an increase of HP1 association at 3R-TAS [21]. Likewise, we observe a mild corresponding increase in HP1 association and silencing at piRNA clusters in *piwi* mutants suggesting that *piwi* function could in fact oppose HP1 recruitment at multiple sites in the genome. Our results are consistent with the possibility that piRNA clusters act as boundaries to the spread of pericentromeric heterochromatin. The mechanism of Piwi dependent transcriptional activation has not been determined, but considering that Piwi interacts with the chromoshadow domain of HP1 [11], Piwi may compete for binding with other HP1 interactors such as Su(var)3–9 that promote heterochromatic silencing.

### Functions for *piwi* outside of the gonad

The majority of Piwi protein is found in both somatic and germline tissues of the gonad, yet *piwi* clearly exerts an effect on non-gonadal somatic tissues as well. RT-PCR analysis shows that *piwi* transcript is readily detectable outside the gonad and in somatic cell lines [11],[12], but Piwi protein is difficult to detect [11]. Nevertheless, mutation of *piwi* suggests important functions for this gene outside of the gonad. For example, *piwi* is essential for viability, and loss-of-function mutants display a variety of phenotypes manifest in various non-gonadal somatic tissues such as demonstrated in this study and others, which show a requirement for *piwi* in pairing-dependent silencing, nucleolar integrity, and chromatin insulator function [47]–[50]. For each of these chromatin related studies, it remains a possibility that even a small amount of maternally deposited Piwi could trigger early events in the oocyte or embryo that persist throughout development, manifesting phenotypes visible in adult somatic tissues.

### HP1 redistribution in piRNA pathway mutants

Our results along with previous studies have demonstrated that HP1 mislocalizes from the chromocenter in a subset of piRNA pathway mutants. We found that polytene chromosomes of *flam^1^* mutants exhibit expanded HP1 chromocenter distribution. This result is intriguing because the *flam^1^* mutation affects a single piRNA cluster on the X chromosome but HP1 spreading to other chromosomes is apparent. A previous study detected spreading of HP1 to euchromatic arms especially in *spn-E* mutants [36], and we confirmed this result albeit to a lesser degree, with spreading being comparable to the extent seen in *flam^1^* mutants. Perhaps the increase of TE expression in RNA silencing mutants can stimulate HP1 recruitment and spreading from the centromere, which contains the highest concentration of TEs. In fact, transcription of pericentromeric repeats stimulates RNAi-dependent heterochromatin formation in fission yeast [51]–[53].

Redistribution of HP1 in RNA silencing mutants may indirectly affect silencing at various heterochromatic locations in the genome. Seemingly inconsistent with HP1 spreading, *spn-E*, *aub*, and *piwi* mutants display decreased silencing of P element transgene arrays such as *DX1* and single insertions at pericentromeric regions on chromosomes 2 and 4 [36]. In our study, we found that mutation of *flam* also results in loss of silencing at *DX1*, which is distant from the *flam* locus. This reduced silencing in *trans* could not be due to posttranscriptional events as there are no shared sequences between *DX1* and the *flam* locus. Therefore, we consider the possibility that there exists a finite pool of HP1 that accumulates at the centromere in *flam* and other RNA silencing mutants at the cost of reduced density and reduced silencing at other heterochromatic regions such as the transgene array, the fourth chromosome, and the telomere. The concept of a limited population of HP1 was suggested previously to explain the finding that the Y chromosome behaves as a suppressor of variegation by acting as a sink for HP1 [43].

### Conclusions

Studies in multiple organisms have identified or suggested alternative mechanisms to RNA silencing for the recruitment of HP1 to chromatin. In fission yeast, overlapping and redundant RNAi-dependent and independent mechanisms of heterochromatin formation have been elucidated. In mouse cells, HP1 localization to pericentromeric heterochromatin was found to be RNase A sensitive suggesting that an RNA moiety may be involved in HP1 recruitment [54]. Our data indicate that heterochromatin can form independently of RNA silencing in *Drosophila*. It will be interesting to determine if any of these alternative mechanisms of heterochromatin formation are conserved throughout evolution.

## Materials and Methods

### Drosophila stocks

Fly stocks were maintained at 25°C on standard cornmeal medium. Lines containing *P{EPgy2}DIP1^EY02625^* and *P{EPgy2}EY08366* were obtained from the Bloomington Drosophila Stock Center, and a line harboring *PBac{PB}c06482* was obtained from the Exelixis Collection at Harvard Medical School. Genomic coordinates of these P-element insertions were confirmed by PCR with primers specific to the P-elements and flanking genomic sequences followed by sequencing. For transcriptional reporter assays, transgenes were crossed or recombined into mutant backgrounds and scored against crosses to *yw^67c23^* as a reference. For ChIP and immunofluorescence, Oregon-R was used as a wild type control. The *y v f mal flam^1^*/*FM3* stock was selected for heterozygous females each generation to prevent mobilization and accumulation of TEs. For the *DX1* variegation assay, *DX1/CyO* was crossed to *y w v f mal flam^1^/FM7c; CyO/Sp* flies or *yw^67c23^*; *CyO/Sp* as a reference.

### Transcriptional reporter and eye pigmentation assays

Eye pigmentation of 40 to 60 adult males six days of age was examined, and representative eye photos were taken. To quantify overall levels of eye pigmentation, the heads of 25 male flies of each genotype were dissected, and eye pigmentation was measured as previously described [36]. Briefly, heads were homogenized in 0.8 ml of methanol, acidified with 0.1% HCl and centrifuged. The absorbance of the supernatant was measured at 480 nm.

### Chromatin immunoprecipitation

Adult fly heads or ovaries were dissected and crosslinked with 1.8% formaldehyde for 20 min at 23°C. Chromatin was fragmented to an average size of 300 bp by sonication and incubated with antibodies overnight at 4°C. Quantitative PCR was conducted on Applied Biosystems Real Time PCR system using SYBR Green incorporation (Affymetrix/USB). Amplicon sizes ranged between 150 and 250 bp. Chromatin was immunoprecipitated with the following antibodies: α-HP1 (Covance), α-Su(Hw), α-Piwi (P3G11, a gift from M. Siomi), and normal rabbit or mouse IgG (Santa Cruz Biotechnology). A recombinant N-terminal His-tagged fusion protein of the N-terminal of Su(Hw) (amino acids 1–218, kind gift of M. Labrador) was purified from *E.coli* on a nickel-agarose column and used to immunize guinea pigs using standard procedures. Similar results were obtained using the C1A9 α-HP1 antibody (Developmental Studies Hybridoma Bank), but lower quantities of DNA were obtained. Fifty to one hundred fly heads and twenty five to fifty ovaries were used per IP. The quantities of target genomic regions precipitated by different antibodies were calculated as percent input based on four-point standard curves constructed from input DNA for each primer set. Standard deviation of each PCR performed in quadruplicate was calculated to determine the error of measurement. Two independent ChIP samples were analyzed, and similar results were obtained. ChIP primers were designed to be unique, detecting only sequences present in the *flam* and *80EF* piRNA loci and verified by *in silico* PCR. All primers (Table S1 and Table S2) were checked for both specificity and efficiency by standard agarose gel electrophoresis and real time PCR respectively. Primers to piRNA clusters amplify in the same DNA dilution range as primers specific to *hsp26* and *yellow* single copy genes compared to high copy TE elements (Figure S8). Primers to the *flam* locus were verified to amplify approximately two-fold more DNA from female compared to male genomic DNA. A detailed version of this protocol is available in Text S1.

### Culture of OSC cell line and siRNA knockdowns

The OSC line was maintained and Piwi siRNA knockdown was performed as previously described [30]. Briefly, 3×10^6^ trypsinized cells were resuspended in 0.1 mL of Solution V of the Cell Line Nucleofector Kit V (Amaxa Biosystems) and mixed with 200 pmol of siRNA duplex. Transfection was conducted according to the manufacturer's protocol using the nucleofector program T-029, and the transfected cells were incubated at 25°C for 48 hrs. Protein knockdowns were verified by Western blotting, and ChIP assays were performed on mock and *piwi* siRNA transfected cells (5×10^6^ cells per IP).

### Immunostaining of polytene chromosomes

Preparation and immunostaining of salivary gland polytene chromosomes was performed as described previously [55]. Primary antibodies directed against HP1 (Covance) and Mod(mdg4)2.2 (generated similarly as in [56]) and Alexa Fluor 488 labeled anti-guinea pig or Alexa Fluor 594 labeled anti-rabbit secondary antibodies (Invitrogen-Molecular Probes) were used. The chromosomes were viewed using a Leica epifluorescence microscope and photographed using a Hamamatsu digital camera.

### DX1 variegation assay

Eye pigmentation of 100 to 200 flies was scored. The scoring of variegation was categorized into five groups: Light, Medium-Light, Medium, Medium-Dark and Dark corresponding to the percentage of pigmented facets. Percentage of flies falling into each category was graphed. Representative eye photos were taken.

## Supporting Information

Figure S1 Schematic representation of the *38C* piRNA cluster on chromosome 2L. Genomic locations of small RNAs and P element insertions. Sequence datasets derived from previous studies were mapped to the genome using Bowtie software allowing two mismatches. Piwi-immunoprecipitated, Aub or AGO3-immunoprecipitated and AGO2-immunoprecipitated reads mapping to multiple locations in the genome are indicated in red (with 2 mismatches allowed) and pink (with 0 mismatches allowed) while uniquely mapping reads are in dark blue (with 2 mismatches allowed) and light blue (with 0 mismatches allowed). Strongly variegating (dark green triangle), weakly variegating (light green triangle), and non-variegating P elements with high expression levels (white triangle) are indicated. *SUPor-P* P elements containing insulator sequences are marked by an “I”. Centromere proximal end is marked by a hollow C. RepeatMasker detected sequences are indicated in black.
(0.80 MB TIF)

Figure S2 *Dcr-2* mutants display increased HP1 chromatin association and increased silencing at piRNA clusters. ChIP at (A) *flam* and (B) *80EF* piRNA clusters in wild type (blue) and *Dcr-2^L811fsX^ /+* (orange) from adult heads with antibodies specific to HP1. Values shown are percent input immunoprecipitated for each primer set normalized to *hsp26*. Error bars indicate standard deviation of quadruplicate PCR measurements. (C) Adult eyes of wild type and *Dcr-2^L811fsX^* mutants carrying a *mini-white* transgene inserted in close proximity to the *flam* piRNA cluster.
(1.64 MB TIF)

Figure S3 Su(Hw) does not associate with chromatin at piRNA clusters in heads. ChIP at (A) *flam* and (B) *80EF* piRNA clusters in wild type with antibodies specific to Su(Hw) (blue) and rabbit normal serum (yellow). Percent input immunoprecipitated is shown for each primer set, and error bars indicate standard deviation of quadruplicate PCR measurements.
(0.25 MB TIF)

Figure S4 HP1 chromatin association levels are increased in *piwi* mutants at piRNA clusters. ChIP at (A) *flam* and (B) *80EF* piRNA clusters in *piwi^1^/+* (light grey) and *piwi^1^/piwi^2^* (dark grey) from adult heads with antibodies specific to HP1. Values shown are percent input immunoprecipitated for each primer set normalized to *hsp26*. Error bars indicate standard deviation of quadruplicate PCR measurements.
(0.65 MB TIF)

Figure S5 HP1 chromatin association levels are increased in *AGO2* mutants at piRNA clusters. ChIP at (A) *flam* and (B) *80EF* piRNA clusters in *AGO2^51B^/+* (light blue) and *AGO2^51B^* (dark blue) from adult heads with antibodies specific to HP1. Values shown are percent input immunoprecipitated for each primer set normalized to *hsp26*. Error bars indicate standard deviation of quadruplicate PCR measurements.
(0.82 MB TIF)

Figure S6 HP1 protein levels in wild type, *flam^1^*, *AGO2^51B^* and *piwi^1^/piwi^2^* fly heads. Total protein was extracted from twenty adult heads by homogenization in RIPA buffer and separated by SDS-PAGE. Immunoblotting of HP1 and Protein on Ecdysone Puffs (Pep), a nuclear protein serving as a loading control, is shown.
(0.43 MB TIF)

Figure S7 *spn-E^hlsE1^*/*spn-E^hlsE616^* mutants display accumulation of HP1 at the chromocenter. Wild type (top) and *spn-E^hlsE1^*/*spn-E^hlsE616^* (bottom) polytene chromosomes stained with antibodies directed against HP1 (green) or a reference protein Mod(mdg4)2.2 (red). DNA is stained with DAPI (blue).
(1.63 MB TIF)

Figure S8 ChIP primer efficiency and specificity. PCR amplification efficiency and specificity of ChIP primers at (A) *flam* and (B) *80EF* piRNA loci are graphed as a function of C_t_ values over DNA concentration. Cycle threshold (C_t_) values of standard curves of input from a representative experiment were graphed to show that primers to piRNA clusters amplify in the same DNA dilution range as primers specific to single copy genes *hsp26* and *yellow* compared to high copy TE elements.
(0.62 MB TIF)

Table S1 Primer set sequences used for ChIP at the *flam* piRNA cluster.
(0.05 MB DOC)

Table S2 Primer set sequences used for ChIP at the *80EF* piRNA cluster.
(0.05 MB DOC)

Text S1 Detailed ChIP protocol.
(0.03 MB DOC)
