# Supplementary figures and images for: HP1 Recruitment in the Absence of Argonaute Proteins in Drosophila
Source: PLoS Genet. 2010 Mar 12;6(3):e1000880. doi: 10.1371/journal.pgen.1000880 (PMC2837403; doi:10.1371/journal.pgen.1000880)

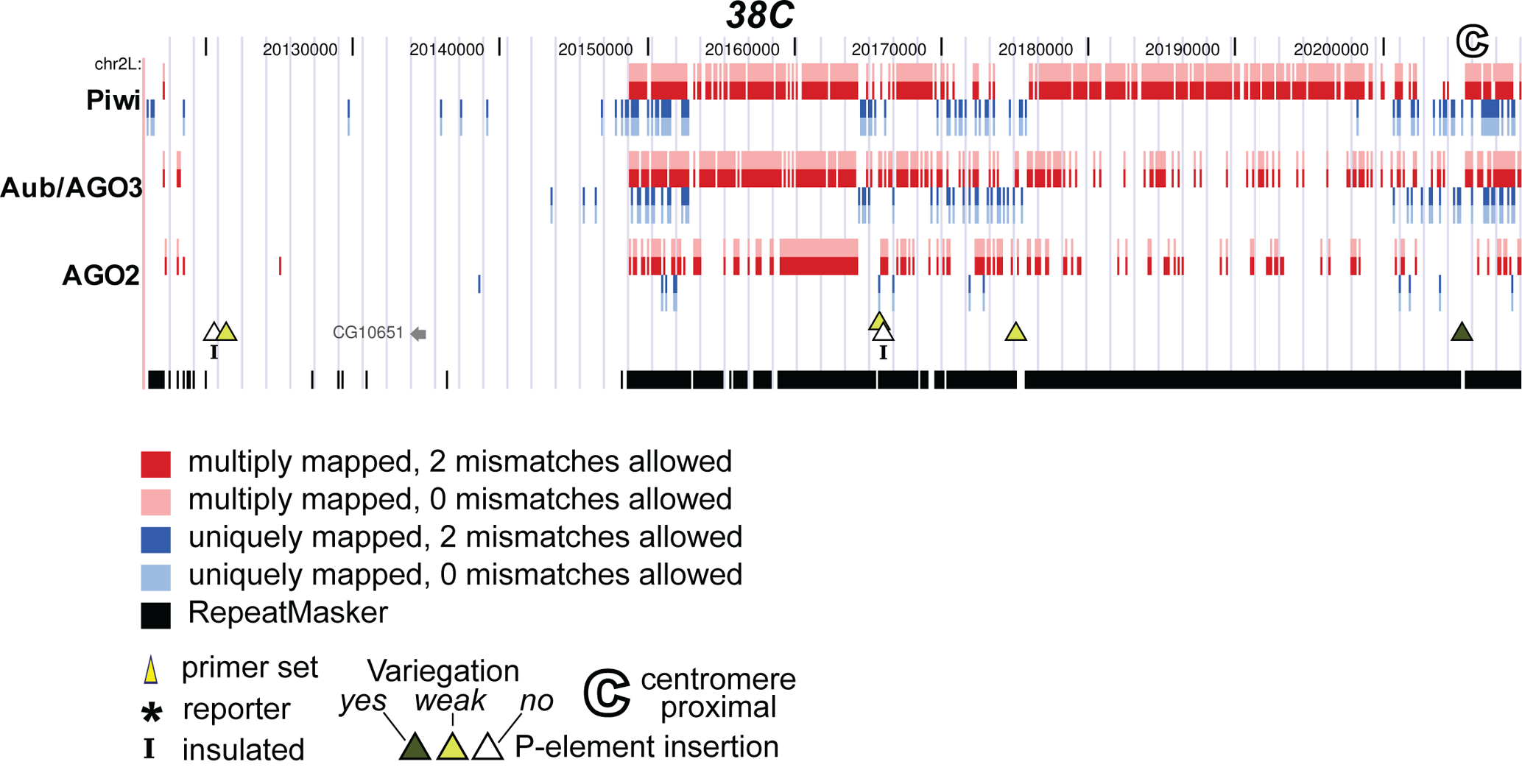

Supplement: Figure S1 — Schematic representation of the 38C piRNA cluster on chromosome 2L. Genomic locations of small RNAs and P element insertions. Sequence datasets derived from previous studies were mapped to the genome using Bowtie software allowing two mismatches. Piwi-immunoprecipitated, Aub or AGO3-immunoprecipitated and AGO2-immunoprecipitated reads mapping to multiple locations in the genome are indicated in red (with 2 mismatches allowed) and pink (with 0 mismatches allowed) while uniquely mapping reads are in dark blue (with 2 mismatches allowed) and light blue (with 0 mismatches allowed). Strongly variegating (dark green triangle), weakly variegating (light green triangle), and non-variegating P elements with high expression levels (white triangle) are indicated. SUPor-P P elements containing insulator sequences are marked by an “I”. Centromere proximal end is marked by a hollow C. RepeatMasker detected sequences are indicated in black. (0.80 MB TIF) [file pgen.1000880.s001.tif]

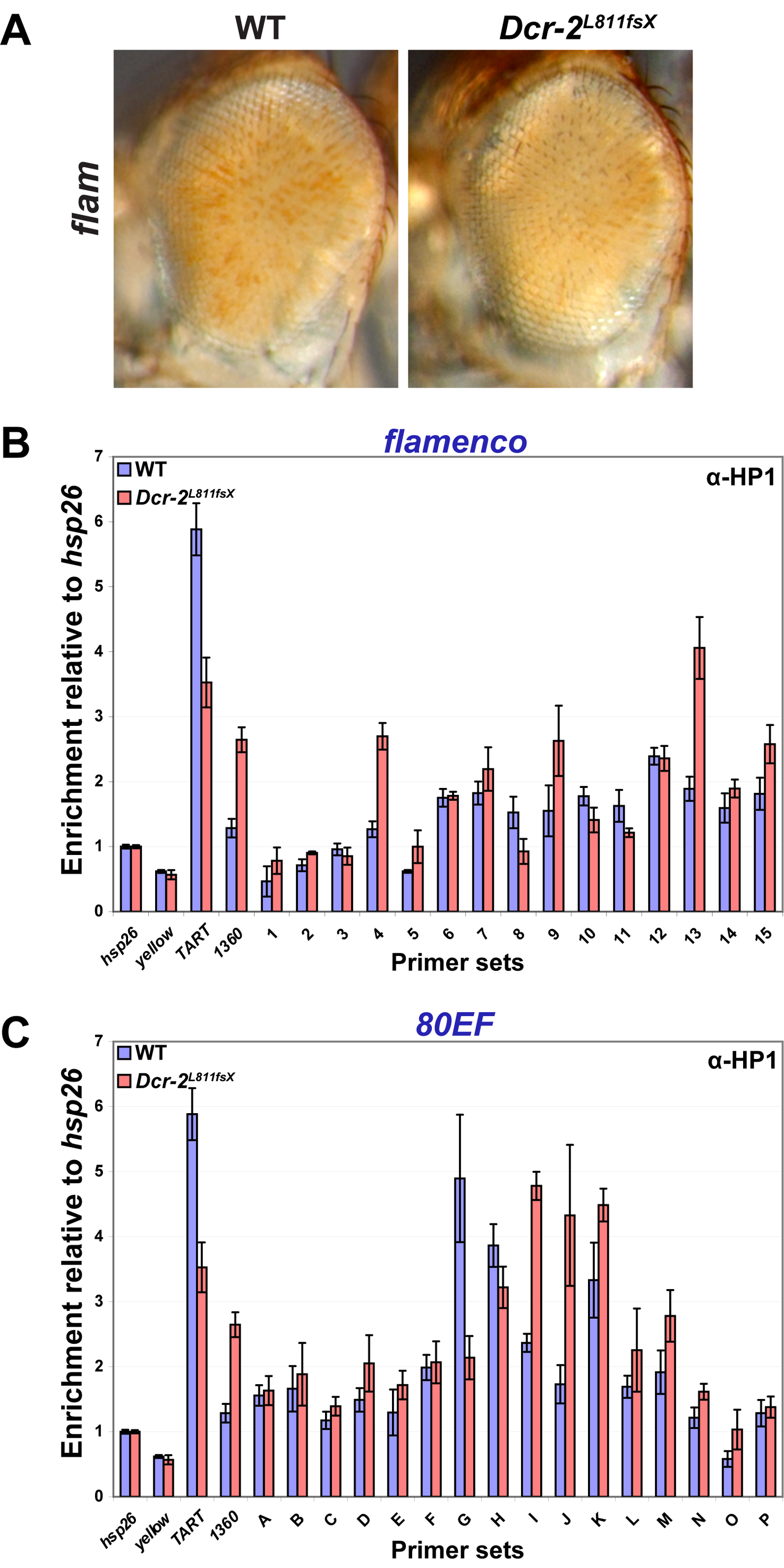

Supplement: Figure S2 — Dcr-2 mutants display increased HP1 chromatin association and increased silencing at piRNA clusters. ChIP at (A) flam and (B) 80EF piRNA clusters in wild type (blue) and Dcr-2L811fsX /+ (orange) from adult heads with antibodies specific to HP1. Values shown are percent input immunoprecipitated for each primer set normalized to hsp26. Error bars indicate standard deviation of quadruplicate PCR measurements. (C) Adult eyes of wild type and Dcr-2L811fsX mutants carrying a mini-white transgene inserted in close proximity to the flam piRNA cluster. (1.64 MB TIF) [file pgen.1000880.s002.tif]

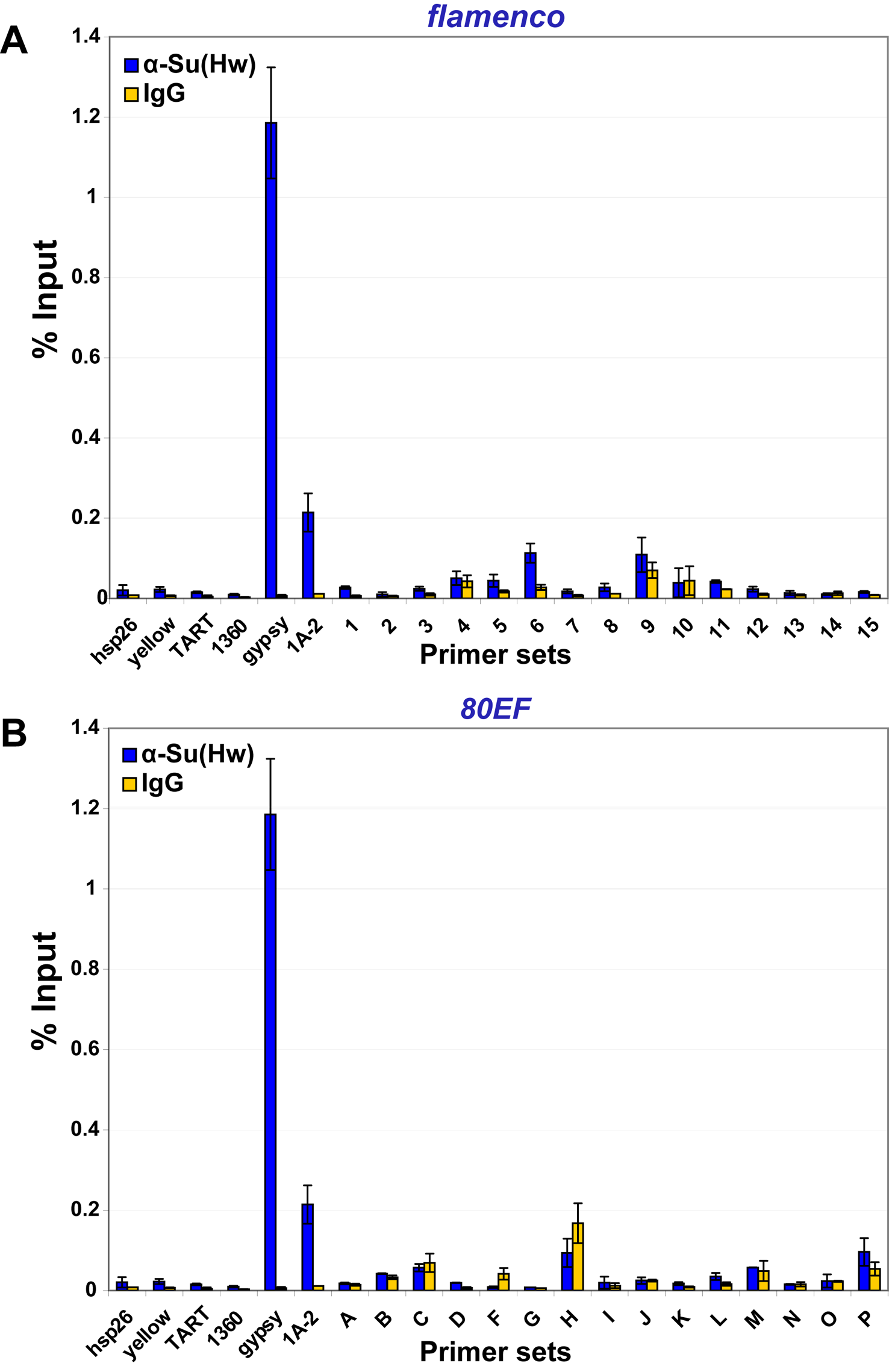

Supplement: Figure S3 — Su(Hw) does not associate with chromatin at piRNA clusters in heads. ChIP at (A) flam and (B) 80EF piRNA clusters in wild type with antibodies specific to Su(Hw) (blue) and rabbit normal serum (yellow). Percent input immunoprecipitated is shown for each primer set, and error bars indicate standard deviation of quadruplicate PCR measurements. (0.25 MB TIF) [file pgen.1000880.s003.tif]

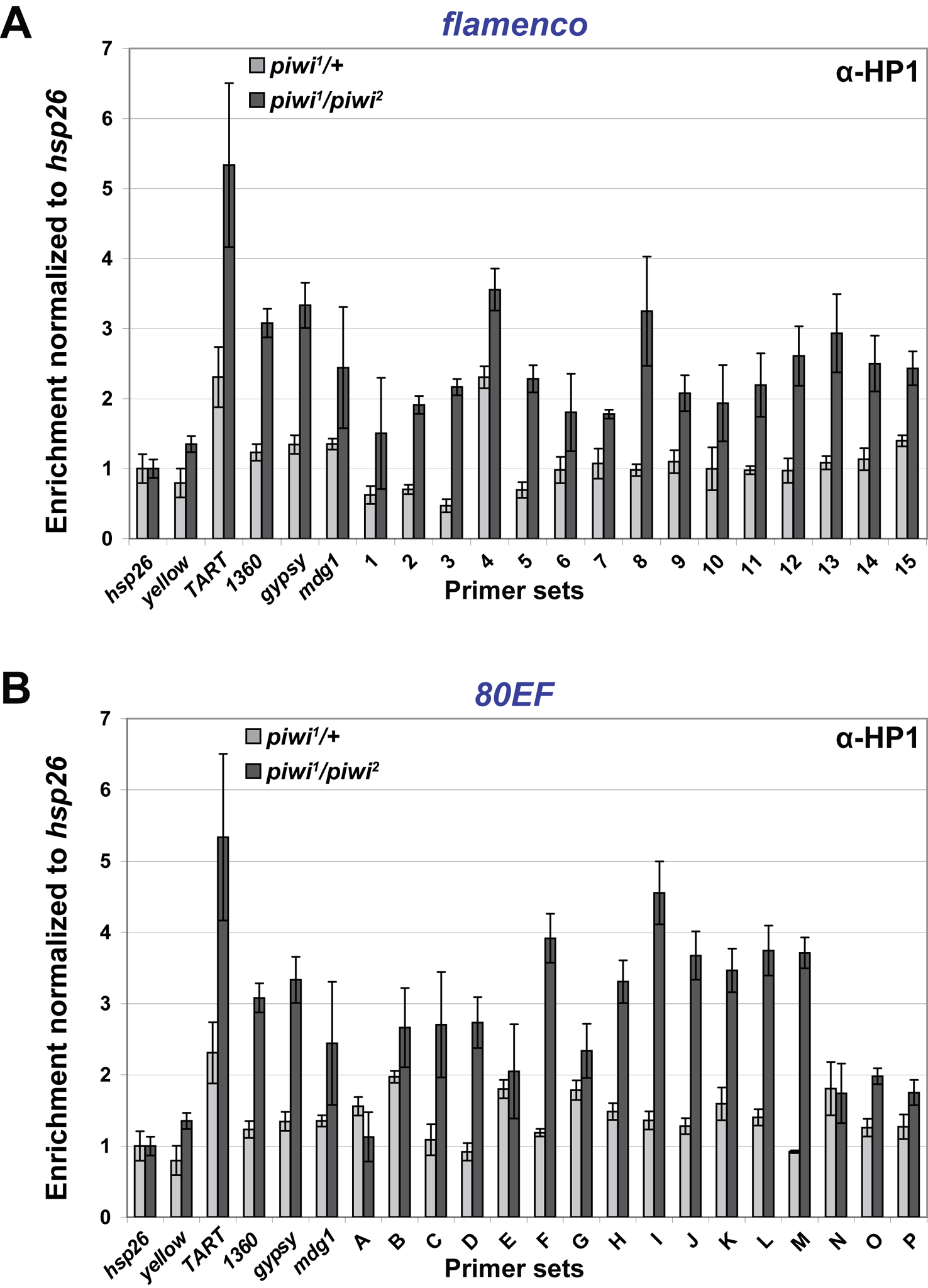

Supplement: Figure S4 — HP1 chromatin association levels are increased in piwi mutants at piRNA clusters. ChIP at (A) flam and (B) 80EF piRNA clusters in piwi1/+ (light grey) and piwi1/piwi2 (dark grey) from adult heads with antibodies specific to HP1. Values shown are percent input immunoprecipitated for each primer set normalized to hsp26. Error bars indicate standard deviation of quadruplicate PCR measurements. (0.65 MB TIF) [file pgen.1000880.s004.tif]

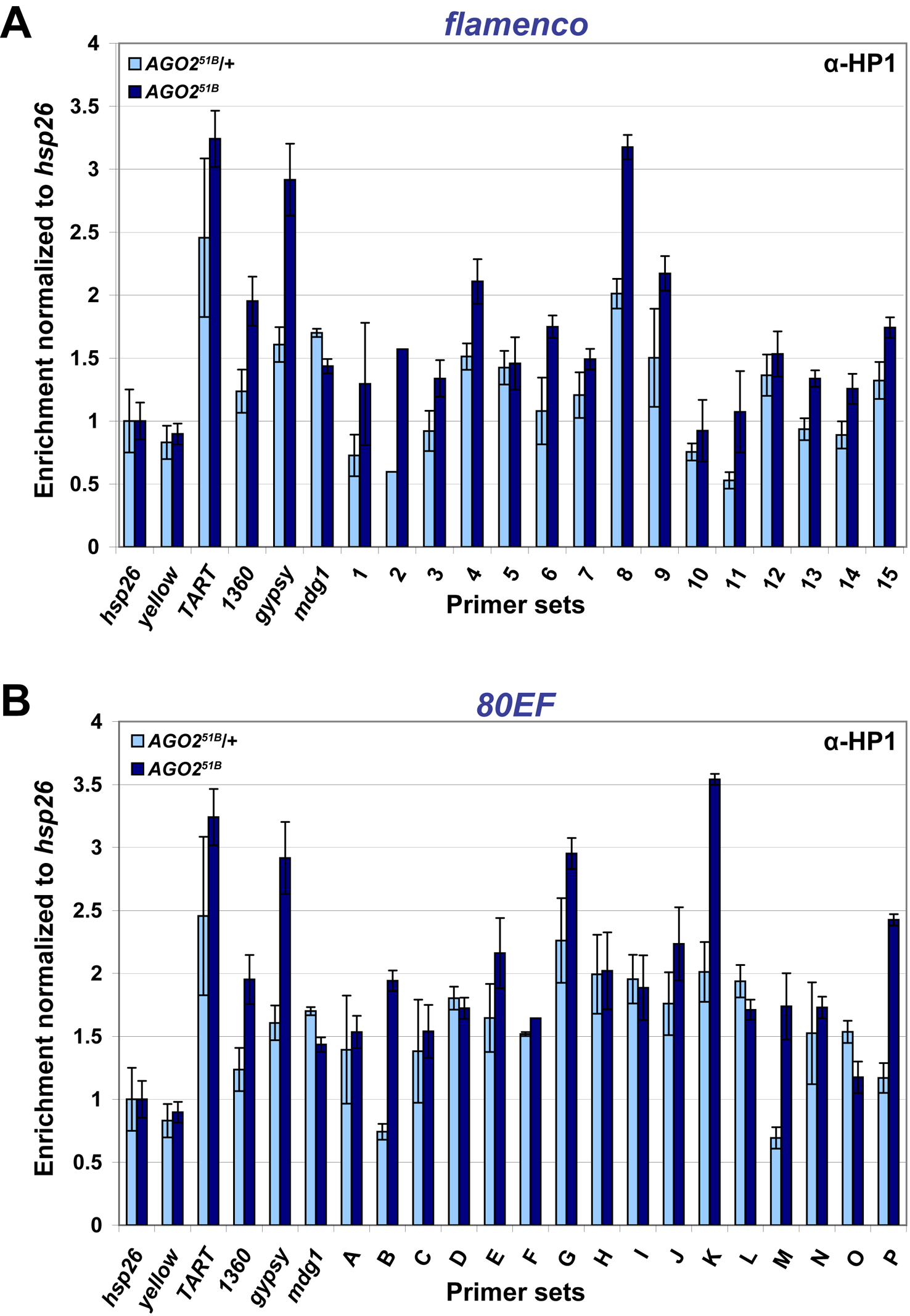

Supplement: Figure S5 — HP1 chromatin association levels are increased in AGO2 mutants at piRNA clusters. ChIP at (A) flam and (B) 80EF piRNA clusters in AGO251B/+ (light blue) and AGO251B (dark blue) from adult heads with antibodies specific to HP1. Values shown are percent input immunoprecipitated for each primer set normalized to hsp26. Error bars indicate standard deviation of quadruplicate PCR measurements. (0.82 MB TIF) [file pgen.1000880.s005.tif]

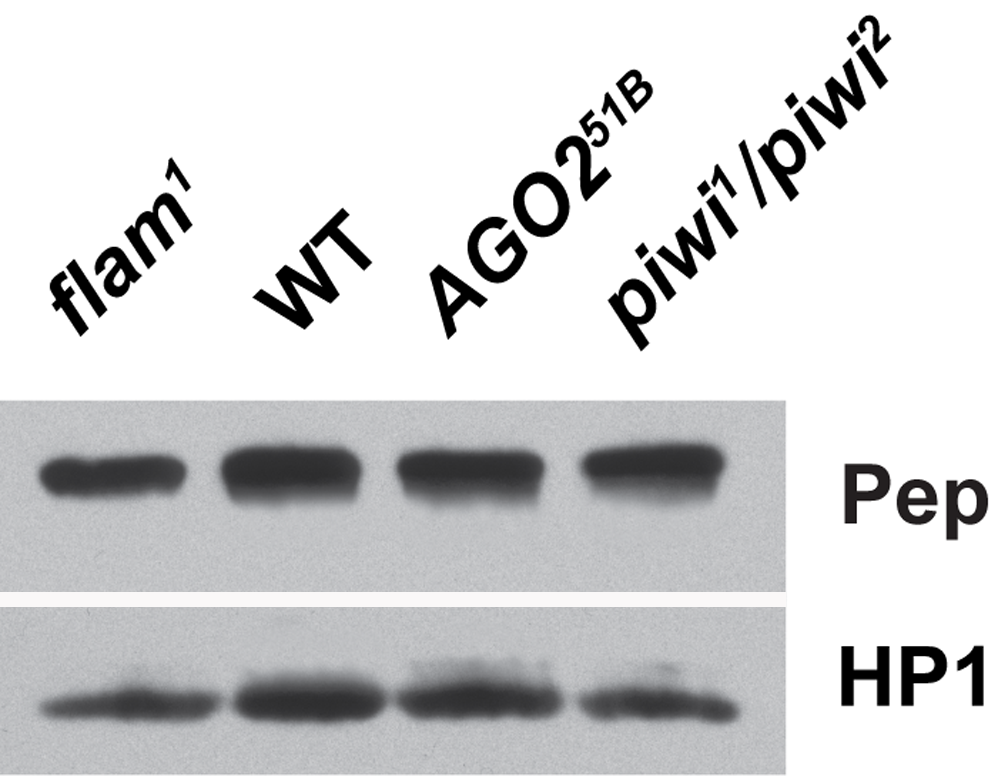

Supplement: Figure S6 — HP1 protein levels in wild type, flam1, AGO251B and piwi1/piwi2 fly heads. Total protein was extracted from twenty adult heads by homogenization in RIPA buffer and separated by SDS-PAGE. Immunoblotting of HP1 and Protein on Ecdysone Puffs (Pep), a nuclear protein serving as a loading control, is shown. (0.43 MB TIF) [file pgen.1000880.s006.tif]

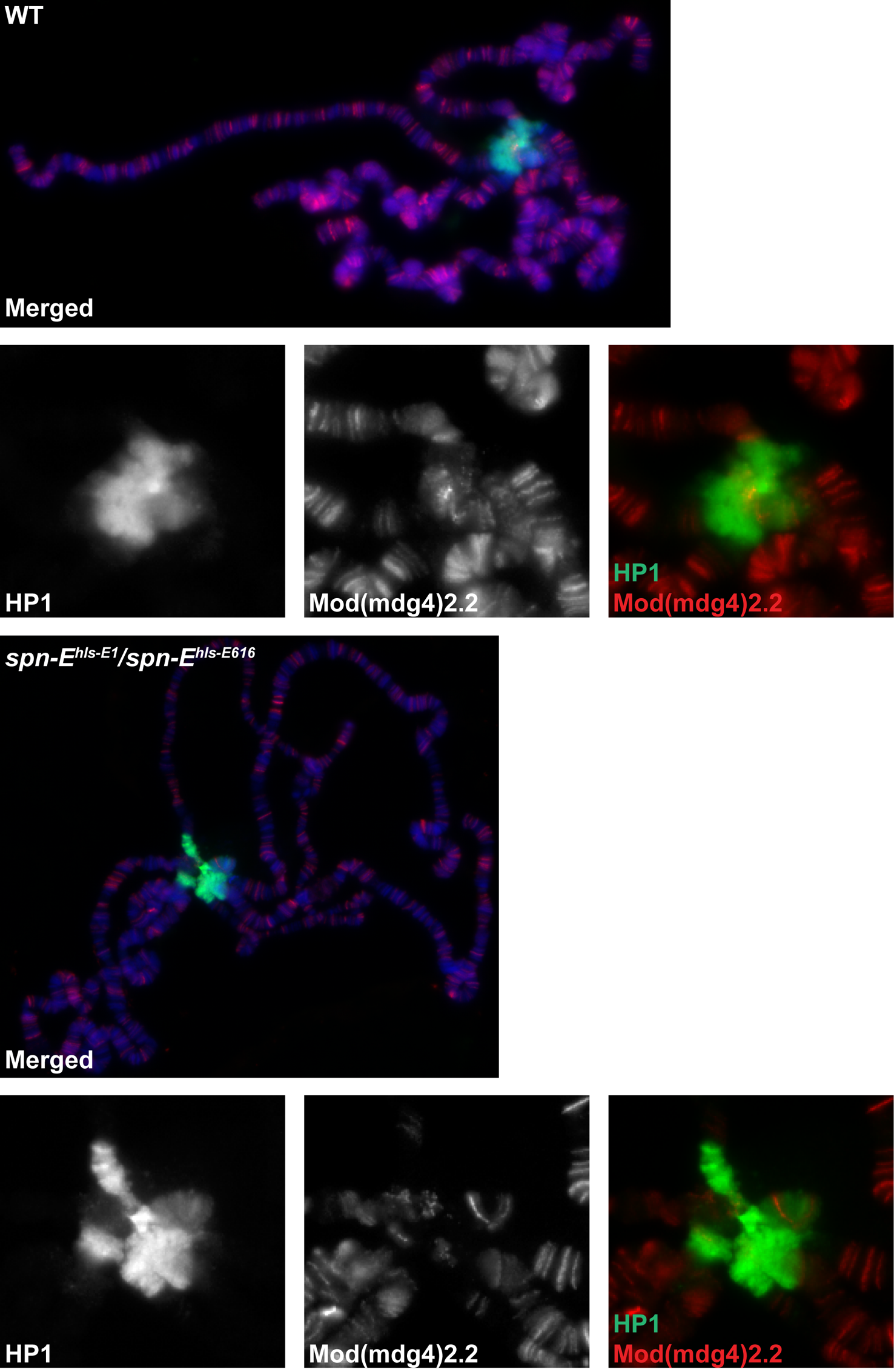

Supplement: Figure S7 — spn-EhlsE1/spn-EhlsE616 mutants display accumulation of HP1 at the chromocenter. Wild type (top) and spn-EhlsE1/spn-EhlsE616 (bottom) polytene chromosomes stained with antibodies directed against HP1 (green) or a reference protein Mod(mdg4)2.2 (red). DNA is stained with DAPI (blue). (1.63 MB TIF) [file pgen.1000880.s007.tif]

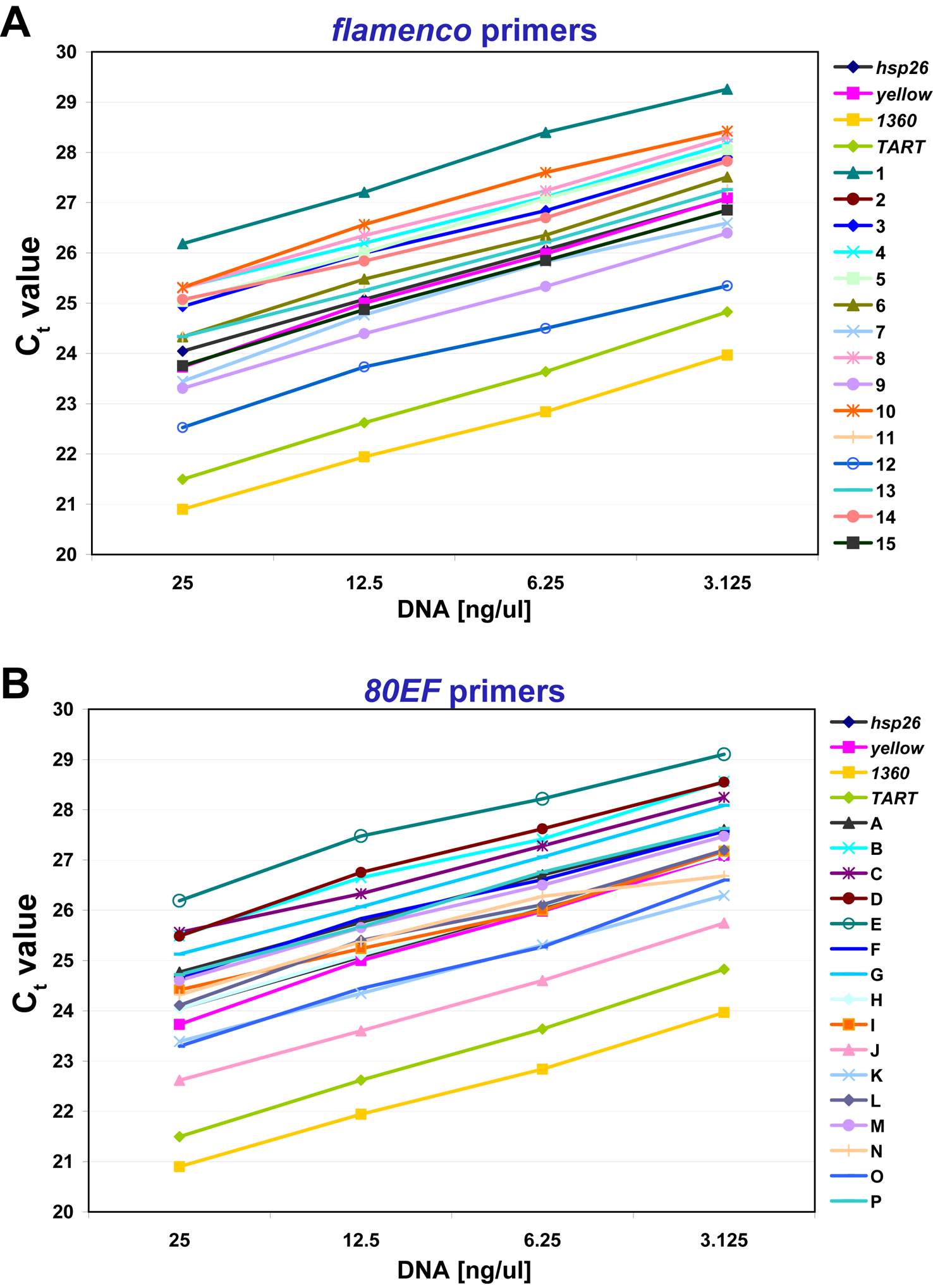

Supplement: Figure S8 — ChIP primer efficiency and specificity. PCR amplification efficiency and specificity of ChIP primers at (A) flam and (B) 80EF piRNA loci are graphed as a function of Ct values over DNA concentration. Cycle threshold (Ct) values of standard curves of input from a representative experiment were graphed to show that primers to piRNA clusters amplify in the same DNA dilution range as primers specific to single copy genes hsp26 and yellow compared to high copy TE elements. (0.62 MB TIF) [file pgen.1000880.s008.tif]
