# Supplementary material for: HP1 Recruitment in the Absence of Argonaute Proteins in Drosophila
Source: PLoS Genet. 2010 Mar 12;6(3):e1000880. doi: 10.1371/journal.pgen.1000880 (PMC2837403; doi:10.1371/journal.pgen.1000880)
Supplement: Table S1 — Primer set sequences used for ChIP at the flam piRNA cluster. (0.05 MB DOC) [file pgen.1000880.s009.doc]

**Table S1**. **Primer set sequences used for ChIP at the *flam* piRNA cluster.**

|  | **Sequence 5’ to 3’** | **Genomic coordinates** |
| --- | --- | --- |
| **1** | cgttcatgtcgttccacaac | chrX:21473147+21473333 187bp |
|  | tgcacggatcgtggttatta |  |
| **2** | aaaccacttcgcggatttc | chrX:21495641+21495810 170bp |
|  | tgcattttgatttcttgtgctc |  |
| **3** | aacgaggccagattcaacat | chrX:21497545+21497751 207bp |
|  | gaatcagtacgagggcaagg |  |
| **4** | caagttggggtttcgtgttt | chrX:21504581+21504730 150bp |
|  | attgaaccttaccccgacaa |  |
| **5** | ggagtgggatggatagacga | chrX:21510544+21510729 186bp |
|  | cctggacacaggaccaaagt |  |
| **6** | ctcgggattttgcgttacat | chrX:21526571+21527094 231bp |
|  | ggcagctaaccgtggataaa |  |
| **7** | gtggcttcacaaaacacgac | chrX:21527157+21527376 220bp |
|  | cgaaggcttacacgcaagat |  |
| **8** | cctaccaacccagcgaataa | chrX:21537802+21538037 236bp |
|  | tgctcttaagcctgcgaaat |  |
| **9** | cgatccgtttatgcaggtct | chrX:21539214+21539437 224bp |
|  | ctgccaacaaatccatttcc |  |
| **10** | tgcctgtcgtactttgcttg | chrX:21543255+21543448 194bp |
|  | ccaatgaattgccgctagtt |  |
| **11** | cgcgactgattggaagaact | chrX:21586797+21586976 180bp |
|  | tctaagcccaacgtacacga |  |
| **12** | tcaggattcctccagaggtg | chrX:21604099+21604347 249bp |
|  | ggccgctatgagtttcatgt |  |
| **13** | tgcgtgacgtaagcaaactc | chrX:21605733+21605922 190bp |
|  | ttttatcggtggtgggaaag |  |
| **14** | cgggtgtaggtcacttggtt | chrX:21611603+21611784 182bp |
|  | cagttaccaacgcaatcacg |  |
| **15** | tgcgtgccttttaaggagtc | chrX:21618001+21618210 210bp |
|  | cgctgaatgcgatagtgaca |  |
