# Supplementary material for: HP1 Recruitment in the Absence of Argonaute Proteins in Drosophila
Source: PLoS Genet. 2010 Mar 12;6(3):e1000880. doi: 10.1371/journal.pgen.1000880 (PMC2837403; doi:10.1371/journal.pgen.1000880)
Supplement: Table S2 — Primer set sequences used for ChIP at the 80EF piRNA cluster. (0.05 MB DOC) [file pgen.1000880.s010.doc]

**Table S2. Primer set sequences used for ChIP at the *80EF* piRNA cluster.**

|  | **Sequence 5’ to 3’** | **Genomic coordinates** |
| --- | --- | --- |
| **A** | aggcacatggatgaacaaca | chr3L:23255817+23255966 150bp |
|  | gtttggttaacgggcaacat |  |
| **B** | accgtgcatcccaatatcat | chr3L:23257001+23257188 188bp |
|  | ccaccaaaagaaagaacacg |  |
| **C** | aggacacacatgcttgctttt | chr3L:23262518+23262618 101bp |
|  | cgataaatcttcttttggcaga |  |
| **D** | tagcattacggcgaatggac | chr3L:23271313+23271532 220bp |
|  | ctctgcaataaagcgcacac |  |
| **E** | gcttcgaagaagtgcaatca | chr3L:23277432+23277632 201bp |
|  | ttttgagcgggttttattcg |  |
| **F** | ggacggtttgtttgtcttcg | chr3L:23278084+23278273 190bp |
|  | gactcgatgtggccatgata |  |
| **G** | ttttgcatgtggcaataatca | chr3L:23281146+23281329 184bp |
|  | cgcatcggatattgtctgtg |  |
| **H** | cgaggcatgtcgtagctgta | chr3L:23290484+23290709 226bp |
|  | gccctagtggcctcttctct |  |
| **I** | cctcattttcgcctcgatta | chr3L:23291884+23292130 247bp |
|  | aaaagaaccgcaagagagca |  |
| **J** | tcgatgagcaagatgtgagg | chr3L:23295139+23295322 184bp |
|  | aaacgagatggccaacaaag |  |
| **K** | agggtccggttctcttctgt | chr3L:23300821+23301000 180bp |
|  | aaaacttggttgccctgatg |  |
| **L** | tcgtggtgcagttgagagtc | chr3L:23307902+23308093 192bp |
|  | aagagcggcagagagtcaag |  |
| **M** | aaatcaaacggagtttctgtttct | chr3L:23308478+23308657 180bp |
|  | caagctcaaagtgccatcaa |  |
| **N** | tttcggaagctggtacaaag | chr3L:23312351+23312521 171bp |
|  | cgccgcttatattttgaacg |  |
| **O** | ctagtttttcagcgtgcttgg | chr3L:23322270+23322429 160bp |
|  | ctaagaaggcaattgcgaaag |  |
| **P** | ggagctattggagccgtcta | chr3L:23332664+23332763 100bp |
|  | tgtactcttgccatggttcg |  |
