# Supplementary material for: HP1 Recruitment in the Absence of Argonaute Proteins in Drosophila
Source: PLoS Genet. 2010 Mar 12;6(3):e1000880. doi: 10.1371/journal.pgen.1000880 (PMC2837403; doi:10.1371/journal.pgen.1000880)
Supplement: Text S1 — Detailed ChIP protocol. (0.03 MB DOC) [file pgen.1000880.s011.doc]

**Supplementary information for**

**HP1 recruitment in the absence of Argonaute proteins in *Drosophila***

Nellie Moshkovich and Elissa P. Lei

**Detailed ChIP protocol**

**Crosslinking and sonication**

Wildtype (Oregon-R) heads or ovaries were dissected on dry ice. Fly heads/ovaries were washed in 5 ml PBS containing 0.01% Triton X-100 and centrifuged for 1 min at 500 rcf to pellet heads. Supernatant was discarded, and 1 ml of crosslinking solution (50 mM HEPES, pH 8.0, 1 mM EDTA, 0.5 mM EGTA, 100 mM NaCl, and 1.8% formaldehyde) and 3 mL n−heptane were added. The mixture was shaken vigorously for 20 min at room temperature. Supernatant was discarded, and heads were resuspended in 5 ml PBS containing 125 mM glycine and 0.01% Triton X-100. The mixture was shaken for 5 min at room temperature. Supernatant was discarded after centrifugation, and 5 ml of ice cold PBS containing 0.01% Triton X-100 was added. Supernatant was removed and heads were resuspended in 5 ml ice cold PBS containing 0.01%Triton X-100 and protease inhibitors (Roche). Heads were Dounce homogenized with pestle A (Kontes) for tissue disaggregation and complete homogenization. The mixture was centrifuged at 400 rcf for 1 min, and supernatant was transferred to a fresh tube. Supernatant was centrifuged at 9190 rcf for 5 min at 4°C and supernatant discarded afterwards. The pellet was resuspended in 5 ml ice cold Cell Lysis Buffer (5 mM PIPES, pH 8, 85 mM potassium chloride, 0.5% Nonidet P40 (NP40) and protease inhibitors). The mixture was Dounce homogenized with pestle B to release the cell nuclei and centrifuged at 9190 rcf for 5 min at 4°C. Supernatant was removed, and the pellet was resuspended in 1 ml of ice cold Nuclear Lysis Buffer (50 mM Tris HCl, pH 8.0, 10 mM EDTA, 1% SDS and protease inhibitors) and incubated for 20 min at 4°C. Then 0.5 ml ice cold IP dilution buffer (0.01% SDS, 1% Triton X-100, 1.2 mM EDTA pH 8.0, 16.7 mM Tris.HCl, pH 8.0, 167 mM NaCl and protease inhibitors) and 0.3 g of acid washed 212−300 micron glass beads (Sigma) was added. The mixture was sonicated in ice water 8 times for 30 s with 30 s intervals, transferred to microfuge tubes and centrifuged at 18407 rcf for 10 min at 4°C.

**Quality control of input chromatin**

100 μl of chromatin was adjusted to 200 μl with IP dilution buffer and decrosslinked at 65^o^C overnight. 2μl of proteinase K at 20 mg/ml (Invitrogen) was added, and the mixture was incubated at 55^o^C for 2 h. Chromatin was extracted twice with equal volume of phenol:chloroform (Sigma) and once with chloroform (Sigma). 2 μl of glycogen and one-tenth volume of 3M NaOAc, pH5.2 and 2.5 volumes of ethanol were added. The mixture was incubated for 30 min at -20°C, centrifuged and washed twice with 70% ethanol. The pellet was dissolved in 50 μl water. Chromatin size was checked on a 1% agarose gel. DNA was quantified using Nanodrop ND1000 spectrophotometer.

**Preparation of Protein A beads**

rProtein A agarose beads (GE Healthcare) were washed with IP dilution buffer 3 times and blocked in IP dilution buffer containing 1% BSA rotating at 4°C overnight. Beads were washed three times in IP dilution buffer and resuspended in IP dilution buffer for later usage.

**Chromatin preclear**

Chromatin was diluted three to five times with IP dilution buffer (depending on the DNA concentration). 30 μl of washed Protein A agarose beads were added to each fraction of diluted chromatin to be used in later immunoprecipitation and incubated at 4°C for 2 h.

**Immunoprecipitation**

Same volume of precleared chromatin was aliqouted for each IP sample. 3-5 μl of antibodies were added to aliquoted chromatin and incubated rotating at 4°C overnight. 30 μl of washed beads previously blocked with IP dilution buffer containing 1% BSA were added to each IP sample. The mixture was incubated at 4°C for 4 h. Chromatin-bound beads were centrifuged at 587 rcf for 2 min, and supernatant was discarded. Beads were washed three times with 1 mL Low salt wash buffer (0.1% SDS, 1.0% Triton X-100, 2.0 mM EDTA pH 8.0, 20 mM Tris.HCl, pH 8.0, 150 mM NaCl), three times with High salt wash buffer (0.1% SDS, 1.0% Triton X-100, 2.0 mM EDTA, pH 8.0, 20 mM Tris.HCl, pH 8.0, 500 mM NaCl), and two times with LiCl buffer (4 M LiCl, 1 mM EDTA, pH 8.0, 10 mM Tris HCl, pH 8.0, 1% NP40, 1% SDC) for 5 min with rotation at RT and pelleted at 587 rcf for 2 min. Chromatin was eluted with 200 μl of freshly prepared IP Elution Buffer (0.1 M NaHCO_3_, 1% SDS) at 65°C for 30 min. This step was repeated one more time. NaCl (5 M, 20μl), EDTA (0.5 M, 8μl), Tris (1 M, pH 8.0, 16μl) were added to 400 μl of eluted chromatin and incubated overnight at 65^o^C. 4 μl of Proteinase K at 20 mg/ml were added for 3 h at 55^o^C. Chromatin was extracted with phenol:chloroform and precipitated as described before. IP samples were dissolved in 50 μl water.
